# From the Backyard to Our Beds: The Spectrum of Care, Attitudes, Relationship Types, and Welfare in Non-Commercial Chicken Care

**DOI:** 10.3390/ani14020288

**Published:** 2024-01-17

**Authors:** Jenny L. Mace, Andrew Knight

**Affiliations:** 1 Faculty of Health and Wellbeing, University of Winchester, Sparkford Road, Winchester SO22 4NR, UK; 2 School of Environment and Science, Griffith University, Nathan, QLD 4111, Australia

**Keywords:** backyard chickens, ex-commercial chicken care, pet chickens, Suprelorin^®^ implant, chicken welfare

## Abstract

**Simple Summary:**

Chickens may be the third most common pet in many countries; however, little is known about their welfare status or the human–chicken relationship. Hence, using 2000+ questionnaire responses, this study collected the most comprehensive and holistic dataset to date on owners’ care-taking practices, owners’ attitudes towards chickens, the human–chicken relationship, and challenges regarding chicken welfare. The most important results included the following: variable care-taking practices; largely positive attitudes towards chickens; mid-level closeness regarding the human–chicken relationship; preventing chickens going to slaughter as a top motivator of chicken care; a majority having not heard of Suprelorin^®^ (a hormonal implant to prevent egg laying); only a significant minority having used Suprelorin^®^ in their hens; a higher prevalence of health conditions/higher number of deaths being found relative to the extant literature; and egg yolk peritonitis emerging as a leading health problem and cause of death. Most chicken carers still consumed chicken meat and eggs. This study and its results should help numerous stakeholders in chicken welfare, including vets, owners themselves, and rehoming charities.

**Abstract:**

Non-commercial chickens may be the third most numerous pets in Western countries. Yet, to date, there is limited research into their welfare or the care-taking practices and attitudes of their guardians. Using a quantitative questionnaire, this study investigated non-commercial chicken owners’ care-taking practices, attitudes, and relationship types with their chickens. Additionally, the study investigated barriers to optimizing non-commercial chicken welfare. Specific questions were asked regarding niche care-taking practices, including the use of Suprelorin^®^ implants. With 2000+ responses, this study found variable care-taking practices, yet largely positive attitudes towards chickens, and a “personal” (though not “close personal”) owner–chicken relationship, as defined by the Owner–Bird Relationship Scale. The Chicken Attitude Scale, Owner–Bird Relationship Scale, and Care Series scores were found to be correlated with each other, with coefficients ranging from 0.176 to 0.543 (*p* < 0.001). “Preventing commercial chickens from going to slaughter” was a key motive for chicken care by 56.1% of respondents, with 69.6% of respondents stating they cared for ex-commercial chickens. This study found a higher prevalence of reported poor health conditions and number of deaths relative to prior studies, and egg yolk peritonitis emerged as a leading health condition and cause of death. Moreover, 68.0% had not heard of Suprelorin^®^ implants, and only 6.3% used implants. Most (76.4%) chicken carers followed an omnivorous diet that includes chicken meat/eggs. The results reinforced previous findings concerning a need for more avian-specialist, locally available, and affordable veterinary care for chickens. Research into Suprelorin^®^ implants, rooster-specific care, and tailored requirements of caring for ex-commercial chickens is recommended.

## 1. Introduction

Non-commercial chickens in the domestic environment have been increasing in popularity over the last few years across the Western world. Current higher estimates include five million pet chickens in the UK, which is based on one million households owning chickens and an average of five chickens per household/owner. This ranks non-commercial chickens as the third most numerous pet in the UK, overtaking rabbits [1,2]. This is excluding fishes, who rank first or second if counted as individuals (e.g., [3,4]). The estimate is even higher in the USA, which has over 85 million backyard chickens, based on 13% of 131.2 million households owning chickens [5,6] and an average of five chickens per household/owner. This figure rivals the populations of cats and dogs [7]. There are also reports of further increases in non-commercial chicken numbers resulting from the COVID-19 lockdown [8], and further increases are expected due to celebrities such as the Duke and Duchess of Sussex promoting chicken keeping, especially regarding ex-commercial chicken care [9]. 

Ex-commercial hens are former commercial laying hens who, in the UK, would otherwise be slaughtered around the age of 1.5 years. It is known that the charity The British Hen Welfare Trust (BHWT) rehomes over 60,000 ex-commercial hens each year, in comparison to around 8500 dogs rehomed by the Dogs Trust [10,11]. There are similar, though less developed, charities in several other countries, such as in France [12]. These figures highlight an ongoing need for more research in non-commercial chicken care to ascertain and advance chicken welfare in domestic environments and to learn more about this specific human–bird and human–animal companion relationship. With the post-COVID-19 context, a cost-of-living crisis in the UK and some other nations at the time of writing, and worsening avian influenza, there is an intensifying need for further research.

To date, most of the empirical studies examining non-commercial chickens have concentrated on biosecurity practices and have been limited in scope, focusing on minimal health measures rather than a full conception of welfare (e.g., [13,14,15,16]). However, there is a growing body of international literature regarding non-commercial chicken care that spans qualitative surveys (e.g., [17]), quantitative surveys (e.g., [18]), and an examination of veterinary records (e.g., [19]). Additionally, an increasing number of works feature terms such as “relationship”, “pet”, or “welfare” in the title of the work (e.g., [20,21]). There is evidence to suggest chickens are not strictly confined to the outdoors, and they, like dogs, are succeeding in crossing over the home boundary. Thus, the term “backyard chickens” is arguably not completely accurate. Danovich [22] (p. 10), for instance, uses the term “chickens at home”. This study used the terms “backyard chickens”, “chickens at home”, “household chickens”, and “non-commercial chickens”, interchangeably. Additionally, this study used the terms “pet” and “companion animal”, as well as “guardian”, “owner”, and “carer”, interchangeably. 

As recommended by this aforementioned growing body of diverse literature on non-commercial chickens, the aim of this research was to add to the growing pool of research regarding non-commercial chicken care/welfare and human–chicken relationships. Specifically, the aim was to collect data on some more niche and hitherto un-investigated care-taking practices, such as the use of the Suprelorin^®^ hormonal implant to initiate a break or cessation in egg laying by hens for health benefits [23]. 

### 1.1. Current Knowledge of Non-Commercial Chicken Care

In terms of health and welfare, in a study conducted by Elkhoraibi et al. [24], 41.4% of chicken owners reported health concerns in their chickens in the last year. A similar figure of 37.5% was found by Souvestre et al. [18]. External parasites (ectoparasites) have repeatedly been reported to be the most common health concern, with a prevalence of 11.4% [24] and 30.9% within a two-year period [25]. Karabozhilova et al. [21] also found that parasites were rated as the most common concern by 91% of chicken owners. There is little consistency in terms of the second and third most common conditions; egg binding and mycoplasmosis were the next most common conditions documented by Karabozhilova et al. [21], whilst sudden deaths and diarrhea were the next most common health concerns documented by Pohjola et al. [25]. Diarrhea was also reported as the third by Elkhoraibi et al. [24]. 

Pohjola et al. [25] documented mortality within the last year: no mortality was reported by 38.3%, <10% by 48.6%, and 10–20% by 8% of the respondents (20–30% and 30–40% each garnered 2.3% and >50% garnered just 0.6%). Elkhoraibi et al. [24] documented even lower mortality, with 64% of respondents stating no mortality within the last year. Elkhoraibi et al. [24] documented predation as the most common cause of mortality, affecting 30% of the respondents. Details on predation were not collected in the other studies.

Pohjola et al. [25] found that only 5.1% of respondents had any birds checked by a veterinarian in the last year. Elkhoraibi et al. [24] reported a similar low use of vets, with just 10.9% having consulted a vet in the last year. Souvestre et al. [18] reported greater vet usage by 23.8% of respondents. Karabzhilova et al. [21] found that 57% of respondents would consult a vet if the need arose. Additionally, Elkhoraibi et al. [24] found that 76.6% of respondents did not report any behavioral problems within their flock; those that did experience behavioral problems reported feather pecking to be the most common problem. 

Overall, Pohjola et al. [25] noted that 95.5% of respondents reported their chicken flocks to be in either excellent (54.5%) or good (41%) health. However, Karabozhilova et al.’s [21] welfare assessment, which is derived from questionnaire responses and based on the Five Freedoms and the Welfare Quality Assessment (Poultry), reported lower levels of backyard chicken welfare: 20% were judged to be in need of welfare improvements, 63.3% were judged to have an acceptable level of welfare, and only 16.7% were judged to have a high level of welfare. 

A study conducted by Singleton et al. [19] was the first nationwide examination of veterinary records for backyard chickens in the UK. Among the clinical cases examined, the study demonstrated a euthanasia rate for chickens (whereby euthanasia was recorded in the case notes) of nearly 30 times that reported for dogs, 15 times that reported for cats, and over 7 times that of the rate reported for rabbits [19,26]. Respiratory disease and non-specific signs/open diagnosis consistently featured in the top three problems reported in veterinary records by Singleton et al. [19], Taggers and Baron [27], and Vaught et al. [28]. However, the other leading problems varied, including wasting [19], reproductive disease and predator attacks [27], and trauma [28]. Vaught et al. [28] reported a survival discharge rate of 45%, whilst Taggers and Baron [27] reported a rate of 59.4%. In contrast, according to Pohjola et al. [25], the most common causes of mortality found during post-mortem (natural deaths or euthanasia) examinations were Marek’s disease (27%) and colibacillosis (17%).

### 1.2. Attitudes towards Chickens and the Human–Chicken Relationship

In terms of attitudes towards chickens in the domestic environment, the evidence thus far suggests that owners hold positive attitudes towards chickens. For instance, between 53% and 73% of respondents have stated they view their chickens as pets or have them as pets [18,21,24,25], with “pet” status usually carrying greater moral status and signifying that an animal is inedible/unkillable other than for genuine euthanasia [29]. For the remaining percentages of respondents in these studies, there may have been a distinct category that their chickens fitted into rather than the false dichotomy of livestock or pet. Evidence from interviews with chicken carers has revealed an “in-between” classification in relation to chickens. For instance, some of Zoubek’s [17] (pp. 244, 271) interviewees reported viewing chickens as neither livestock nor pets or as both. The phrases “lower pet status”, “pets with benefits”, and “productive backyard pets” have also been provided by Macauley [30] (p. 14) and Demetriou [20] (p. 65), respectively. 

Connected to this is the unique contradiction present for many pet chicken owners, which Macauley [30] (pp. 35–36) outlined as follows: owners who love their own chickens and would not dream of killing or eating them may continue to consume chicken meat bought from shops. This paradox is also observed by Zoubek [17] (p. 281), who has called for more research into these differences in attitudes towards pet chickens versus other chickens. Some scholars have suggested that this allyship with backyard chickens is subversive as it challenges the dominance of factory farming (e.g., [20]), whilst others (e.g., [31]) have contended the increasing popularity of chickens in the domestic environment potentially only furthers their exploitation.

The aforementioned qualitative work provided valuable contributions; however, this work has focused on general attitudes and approaches to backyard chickens and not specific attitudes towards chickens and their treatment and use more generally. Sample sizes have also been limited. This is where quantitative work can play a role in scaling up a grasp of owners’ attitudes in terms of using validated and specific attitude scales, such as the Animal Attitude Scale [32]. This study sought to apply this scale to chickens for the first time. 

There is a known link between attitudes and relationships. For instance, Robertson et al. [33] demonstrated how a stronger attachment to pets is positively correlated with positive attitudes towards nonhuman animals. Nevertheless, the concept of relationship involves numerous other parameters too, including interaction (or a lack thereof), support, and empathy/attentiveness, as outlined by Burmeister et al. [34]. A plethora of studies have examined the human–animal relationship concerning conventional companion animals, predominantly dogs, cats, and horses. Only very recently, however, has the human–companion animal relationship begun to be examined for less conventional companion animals (e.g., see [35]). Accordingly, Burmeister et al. [34] devised a validated Owner–Bird Relationship Scale (OBRS) comprising four dimensions of human–bird relationships (bird as a human, social support, respect/empathy, and bird reciprocity). This OBRS summarizes three types of human–bird relationships: impersonal, personal, and close personal. This study was one of the first to apply the OBRS specifically to one species—non-commercial chickens in the domestic environment. 

### 1.3. Research Questions

To date, this aforementioned research has pointed to considerable welfare concerns in terms of non-commercial chickens, especially relative to other pets. Alongside calls for more general research (e.g., [19,36,37]), there has been extremely limited focus on the burgeoning practice of ex-commercial chicken care. This study aimed to begin to fill this void. This study also sought to investigate emergent practices within the chicken-caring sphere, including the use of the Suprelorin^®^ hormonal implant, which is both scientifically and anecdotally evidenced to enhance hen welfare, health, and longevity [23,38,39]. To the authors’ knowledge, to date, there have been no empirical studies regarding non-commercial chicken owners’ use of this implant. Nevertheless, it is increasingly mentioned in theoretical explorations of chicken welfare (e.g., [17,40,41]), as well as in some rehoming bodies’ healthcare guidance (e.g., see [42]). 

Accordingly, the research questions for this project were as follows:

1. What chicken care-taking practices and attitudes towards chickens do different kinds of chicken carer have?
2. What type of relationship do different kinds of chicken carers have with their chickens?
3. Is there a relationship between the care-taking practices, the human–chicken relationship, or attitudes towards chickens?
4. What are the biggest challenges owners experience in safeguarding chicken welfare?

## 2. Materials and Methods

### 2.1. Research Design

The decision was made to utilize a quantitative method for this research to complement the qualitative work that has already been carried out in relation to the subtopics of this research regarding human–chicken relationships and attitudes towards the treatment and use of chickens. More quantitative studies are also still required regarding care-taking practices for chickens. Using online convenience sampling, a quantitative questionnaire (see Supplementary Materials A) was promoted on various chicken-oriented Facebook groups such as Official Backyard Chickens BYC (514,000 members, international), Ex Battery Hens Forum (11,400 members, international), and Vegans with Chickens (5600 members, international) from 14 May to 1 December 2021. Purposive sampling in both general and specialist interest groups was used with the intention of collecting sufficient data from particular groups, including male chicken carers, BIPOC (black, indigenous, and people of color) chicken carers, general chicken carers, ex-commercial chicken carers, and vegan chicken carers. The charity BHWT and the UK-registered company ChickenGuard (Cambridgeshire, UK) also promoted the survey to their supporters, clients, and followers during this time. The decision was made to focus specifically on “non-commercial chicken care” in this study to gain insight into the care culture amongst those not influenced by profit. Accordingly, the questionnaire only sought responses from those specifically engaged in non-commercial chicken care, which was made explicit to respondents and defined as not generating any income from the practice.

Online Surveys was the survey platform used by 88% of UK universities in 2019 [43], including our institution, the University of Winchester; accordingly, it was used for this study. A mixture of dichotomous and multiple-choice questions was asked along with one Likert series (the Care Series). Two Likert scales were also utilized. The first was the validated OBRS, as per the work of Burmeister et al. [34]. This was used in an identical format apart from the references to “bird” being replaced with “chicken”, and “it” with “his/her”—alterations that were sanctioned by the original creators of the scale [44]. The second was an adapted form of the validated 10-item Animal Attitude Scale (AAS) by Herzog et al. [32]. It was adapted insofar as it was tailored specifically to chickens. This often meant simply substituting “animals” or a different named species for “chickens” (see questionnaire in Supplementary Materials A for details of the full scale). Hereafter, this scale is referred to as the Chicken Attitude Scale (CAS).

There were three open questions. Additionally, when participants selected “other”, they were offered the opportunity to expand on this answer option using free text. Across 60 questions, there was a mix of dichotomous, categorical, ordinal, interval, ratio, and qualitative data produced. This project did not ask about care-taking practices in relation to chicks, only those relating to adult chickens from the point-of-lay onwards were asked for. This is because most chicken carers acquire their chickens at this point in a chicken’s lifecycle and do not breed chickens [24]. It also served to minimize the length of the questionnaire. 

The length of the questionnaire mirrored other successful surveys regarding chickens in domestic environments, such as the 56-question survey used by Elkhoraibi et al. [24], which generated over 1400 responses. Using an online sample size calculator [45] and adopting a 3% margin of error and 99% confidence interval, the target number of responses was at least 1849, assuming an exemplar world chicken carer population of 80.5 million. This estimated world chicken carer population was arrived at by first determining one Western country’s estimated number of chicken owners (households), such as the UK, where it is 3.5% [1]. Then, an estimated number of worldwide households was determined to be 2.3 billion [46]. Then, 3.5% of 2.3 billion was calculated. The figure of 80.5 million owners of non-commercial chickens is likely an overestimation, as many of these will likely be caring for chickens in a commercial capacity, albeit on a small scale, especially in developing countries. Nevertheless, the only ramification of this was that a larger sample size requirement was calculated than may have truly been necessary, which would only have increased the reliability of the present study.

### 2.2. Analysis

Descriptive statistics were used for the demographic data and the initial presentation of the basic results. Inferential statistics were used when looking for relationships between different variables or significant differences between groups. The language orientation of six items on the CAS and two items in the OBRS needed to be made positive so that all statements were measured in the same direction. Thus, the answer codes of all respondents for these altered statements then needed to be reverse-coded to match the intended rating of the respondents. Following the method applied by Burmeister et al. [34], who devised the OBRS scale, principal components analysis (PCA) with varimax rotation was applied to the OBRS to reveal its four dimensions and subscales, and the Kaiser–Meyer–Olkin Measure of Sampling Adequacy was used to ascertain reliability of its subscales. Cronbach’s alpha was computed for all (sub)scales, and the mean scores and standard deviations for each respondent in each (sub)scale were computed.

The Shapiro–Wilk test affirmed that the mean scores of all subscales for the OBRS as well as the mean score for the CAS and Care Series, were not normally distributed. Thus, despite the scale data being treated as interval data, non-parametric tests were used in the statistical tests. For instance, Spearman’s rho was used to test for any correlation between the scale and ordinal variables [47]. Additionally, Kruskal–Wallis and Mann–Whitney U tests were used to test for significant differences between three or more groups or two groups, respectively. For the Kruskal–Wallis tests, the Bonferroni post hoc test was also used to identify between which groups differences were found [48]. Statistical tests concerning any associations between solely categorical data used the chi-squared test of independence [48]. In such cases, Cramer’s V statistic was used to demonstrate the strength of any association found, with 0 indicating no association, 0.1–0.2 indicating a weak association, 0.3 indicating a moderate association, and over 0.3 indicating a strong association, in accordance with Marchant-Shapiro [49]. In circumstances where the assumptions of the chi-squared test were violated, the likelihood ratio test was used instead [50].

To answer research questions based on “different types of chicken carer”, human demographic variables were partially used to differentiate between chicken carers and also diet type and chicken demographic variables. Additionally, other care styles were also used, such as whether a vet would be consulted in the event that a chicken was unwell. This study focused on the variables that Zoubek [17] found distinguished between the four chicken carer types that she identified in her qualitative study. For instance, Zoubek’s chicken carer category of “pet keeper” was reflected in the “number of chickens”, “vet use”, and “name chickens”. The qualitative responses to open questions were quantified by identifying key themes and tallying their frequency of occurrence. The qualitative elaborations upon the “other” answer option were examined using the eyeballing (visual inspection) method [51].

### 2.3. Research Ethics

All participants were able to remain anonymous. Participants were offered the chance to hear about the results of the study; those wishing to be informed were asked for their email addresses on a separate page, and this was disconnected from the preceding information given. All participants signed a consent form regarding the use of the information they provided in this study. The participants were informed that they could withdraw from participating in the study at any point prior to its completion (see Supplementary Materials A for the information and consent form). The data were stored on a university-provided, secure, password-protected file hosting service (OneDrive), and a password-protected laptop was used. Ethical approval was secured from the University of Winchester on 5 May 2021.

### 2.4. Pilot Study

The questionnaire was piloted on 10 individuals: a mixture of animal welfare academics who kept chickens, lay people who kept chickens, the founder of the BHWT, and a staff member at ChickenGuard. Based on the feedback received, several changes were made, including the following:

- The human demographic questions were relocated to the end of the questionnaire.
- Explicit instructions for respondents were added regarding which chicken to consider when answering the OBRS [34]–namely, the chicken the respondent felt closest to.
- All mentions of “bird” in the OBRS [34] were changed to “chicken”.
- Definitions for “coop” and “run” were added.
- More options were added to some questions, and some questions were altered to enable the selection of multiple options.
- Minor typographical and syntactical errors were corrected.

## 3. Results

The data were transported into IBM SPSS Statistics ver. 27 for analysis. There were 2059 questionnaire respondents. In questions intended for all respondents, the lowest number of responses was 1961. The response rate is unknown as it is unknown how many people saw the survey link each time it was posted on the various platforms. Human and chicken demographic results are reported first, followed by results concerning each of the research questions in turn. Further detail is also provided in the Supplementary Materials, as indicated. Note that the respondents could select multiple answers for several questions; hence, in some cases, the totals exceed the maximum number of participants.

### 3.1. Human Demographics

The vast majority of respondents identified as female (86.9%, n = 1779), followed by male (11.8%, n = 241). Only five (0.2%) respondents identified as “non-binary” and twenty-two (1.1%) selected “rather not say”. Over 60% of the respondents were highly educated, with over 36% (36.6%, n = 750) selecting “undergraduate degree” as their highest education level, and another 25.8% selecting a postgraduate degree (n = 529). Over 95% of respondents were resident in the UK (95.6%, n = 1955). Quantification of the qualitative ethnicity question (answered by 1922 respondents) revealed that the vast majority of respondents identified as white (79.7%, n = 1532), followed by mixed race (1.4%, n = 26). Other ethnicities were given by nine respondents (0.5%), including Hispanic, Asian, Arab, Native American, and Black. A further 354 respondents (18.4%) gave a non-ethnicity answer as their response (e.g., a region or nationality). 

The most common age groups amongst the respondents were 45–54 and 55–64, with an almost identical share of the respondents at 30.2% (n = 621) and 30.1% (n = 618), respectively. The next most common age groups were 35–44 and 65–74, with an almost identical share of the respondents at 14.8% (n = 303) and 14.5% (n = 298), respectively. The least common age groups amongst the respondents were 18–24 (0.7%, n = 15) followed by 74+ (2.1%, n = 43).

The most common living environment selected was rural (39.1%, n = 800), followed by semi-rural (33.1%, n = 677). Just over 6% of the respondents (6.3%, n = 128) selected “urban”, and just over 21% (21.6%, n = 441) selected “suburban” for their living environment. Moreover, just over half lived in a detached house (56.2%, n = 1147), and the vast majority owned their home (92.0%, n = 1860). Only 8.0% of the respondents (n = 161) rented their home. Additionally, there was a broad selection of different garden/land sizes (small, medium, large, and very large), with “large” having been selected most commonly by just over 40% of the respondents (40.6%, n = 829). The respondents were not asked for precise land/garden dimensions. The most common living situation selected was “married/living with partner with no children (they live elsewhere/are grown)” (28.0%, n = 570), followed closely by “married/living with partner with no children (do not have any)” (26.7%, n = 543) and “married/living with partner with children” (26.1%, n = 532).

Nearly half (47.9%, n = 977) of the respondents selected “professional” from the occupational choices. “Retired” was the next most common answer option (24.2% of the respondents, n = 494). The vast majority of respondents care for other nonhuman animals besides chickens, also in a non-commercial capacity. Only 12.9% of the respondents (n = 264) selected “no” to indicate that they care for no other animals. The most common other animal to be cared for was dogs, selected by over half of the respondents (55.2%, n = 1128), followed closely by cats (selected by 47.7% of respondents, n = 974).

**Figure 1:**
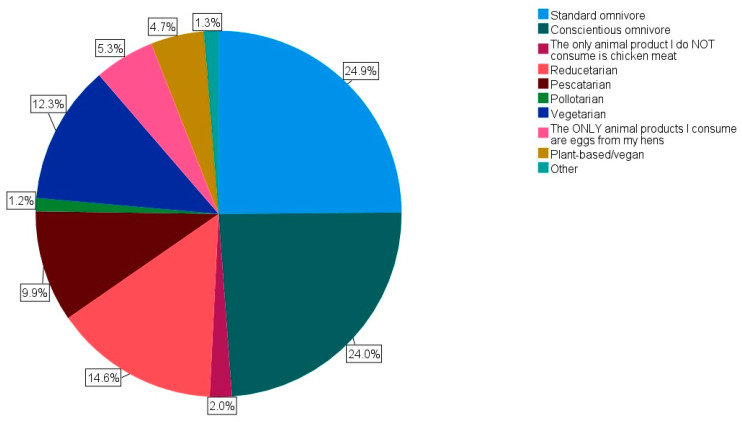
The respondents’ dietary categories are shown (n = 2050). Note: The following descriptions of dietary terms were included in the questionnaire: standard omnivore (“I eat animal products as part of most meals”); conscientious omnivore (“I only purchase animal products from local and higher-welfare sources”); reducetarian (“I am reducing my consumption of animal products”); pescatarian (“I do not eat meat, but I do eat fish and/or seafood”); pollotarian (“I do not eat red meat, but I do eat white meat”); vegetarian (“I do not eat meat, fish, or seafood, but I consume dairy and/or eggs or products containing these”); and plant-based/vegan (“I do not consume any animal products”).

Figure 1 portrays the dietary categories selected by the respondents, with “standard omnivore” and “conscientious omnivore” comprising the two most common dietary categories at 24.9% (n = 510) and 24.0% (n = 491), respectively. These figures create the combined categories of “omnivore” (76.4%, n = 1566), “vegetarian” (17.6%, n = 361), “plant-based/vegan” (4.7%, n = 97), and “other” (1.3%, n = 26). 

In summary, an average respondent could be described as follows: a white female professional, British, aged between 45 and 54, university-educated, a homeowner living rurally in a detached house with a large garden, living with her spouse/partner without children, a guardian of other animals, and following a “standard omnivore” diet.

### 3.2. Chicken Demographics

In this study, 83.4% (n = 1714) of the respondents had just one flock of chickens in their care; however, over 10.5% (n = 217) had two or three flocks in their care, and 6.0% (n = 124) had over three flocks. The two most common number ranges of chickens to care for were 3–6 chickens (56.1%, n = 1155) and 7–12 chickens (25.0%, n = 515). Almost 5% (4.7%, n = 98) cared for just one or two chickens. Over 60% of the respondents (60.5%, n = 1245) stated that the majority of their chickens were ex-commercial hens spared from slaughter. The next most common means of acquiring chickens amongst the respondents was from a local breeder (25.2%, n = 519). “Other” was also selected by 56 respondents (2.7%). This pattern remained when respondents were asked about the origins of all chickens in their care; that is, the most commonly selected option was “ex-commercial hens spared from slaughter” (selected by 69.6%, n = 1429), followed by “from a local breeder” (selected by 42.5%, n = 872). 

Over 80% (80.8%, n = 1654) of the respondents cared solely for hens, 19.1% (n = 390) cared for both roosters and hens, and only 0.1% (n = 3) cared solely for roosters. In this study, ‘roosters’ may refer to roosters, cocks, and cockerels. The most frequent reason selected by respondents (63.9%, n = 1057) as to why they cared solely for hens (rather than in addition to roosters) was due to noise concerns amongst neighbors. Hybrid/ISA Brown was the most common breed/type of chicken cared for, which was selected by 69.9% of the respondents (n = 1431). The next most common breed/type was bantam, which was selected by 340 respondents (16.6%).

In terms of duration in respondents’ care, 30.7% (n = 633) of respondents had been caring for chickens for under three years. A similar proportion of respondents (34.4%, n = 707) had been caring for chickens for nine years or longer. The top two reasons selected for why the chickens were in the respondents’ care were to source higher welfare eggs (56.4%, n = 1161) and to prevent commercial chickens from going to slaughter (56.1%, n = 1154).

In summary, an average chicken described by respondents was an ex-commercial laying hen, acquired through a rehoming charity, part of a flock of 3–6 chickens, a hybrid/ISA Brown breed, kept for reasons of securing higher welfare eggs or preventing commercial laying hens going to slaughter, and in the respondents’ care for less than three years. More details can be found in Figures S1–S6 in Supplementary Materials B.

#### 3.2.1. Ex-Commercial Chickens

Of the 69.6% of respondents (n = 1429) stating that the majority of the chickens in their care were ex-commercial chickens, 90.3% (n = 1289) stated that the original commercial use of their chickens was for eggs. A further 5.2% (n = 74) stated the original use as being both meat and eggs, and 4.5% stated they were unsure about what the original use would have been. Just 0.1% (n = 1) stated that the original use was solely for meat. Additionally, over half (52.1%, n = 743) selected “cage (enriched or unenriched)” as the farm type from which their chickens came, whilst 20.4% (n = 291) were unsure of the farm type. Just 2.2% (n = 32) selected “organic” as the farm type, 11.4% (n = 162) as “barn”, and 13.9% (n = 199) as free range.

When asked how they acquired their ex-commercial chickens, 97.1% (n = 1383) selected “through a rehoming charity”. Only 0.8% (n = 8) selected “through the Animal Save movement”, and 0.1% (n = 1) selected “through rescuing some from a truck of chickens at a road traffic accident”. However, 5.0% of the respondents (n = 71) selected “other”, which included independent rescue from a commercial setting and direct acquisition from local farms. 

#### 3.2.2. Indoor and Disabled Chickens

The vast majority (87.9%, n = 1793) of respondents had not cared for a chicken who lived primarily indoors in owners’ homes. Additionally, it was found that 60.7% (n = 1084) would never consider this. From 1068 qualitative (free text) responses, common reasons for this included the following: difficulty in managing other pets, difficulty in managing chicken feces, perceptions of needing outside space for welfare (temperature and natural behaviors), and a partner not agreeing.

Similarly, a large majority (80.3%, n = 1645) had not cared for chickens with special needs or who were disabled. Many respondents (98.8%, n = 398) of the 19.7% (n = 403) who had experienced caring for a disabled chicken shared some cases of this. Recurring examples included the following: use of chicken wheelchairs/wheeled walking frames (broken legs); caring for blind hens; syringe-feeding; medicating for two years (heart problem); epilepsy; broken wings; deformed beaks, leading to feeding support; dislocated hip joint; wry neck; nebulizing chickens for 30 min twice a day due to aspergillosis; and broken back leading to the use of a hammock or, in another case, cortisone shots and full recovery; broken legs and the use of a splint; daily medications for egg yolk peritonitis (EYP) or fluid retention problems; relearning after a stroke; amputations of feet following fox attacks; and deaf chickens.

It was found that 57.1% (n = 939) of the 80.3% who had not cared for a disabled chicken would be willing to care for a disabled chicken; a further 30.7% (n = 505) stated they were unsure if they would be willing, whilst 12.2% (n = 201) stated they would not be willing. The main reasons for selecting “no” included concerns regarding the quality of life of the disabled chickens and having insufficient time/experience/space.

### 3.3. What Chicken Care-Taking Practices Do Chicken Carers Have?

#### 3.3.1. General/Chicken Living Quarters

Nearly 80% (70.9%, n = 1459) of the respondents stated they were the primary carer for their chickens, whilst over 26% (26.5%, n = 546) stated they shared chicken care duties 50:50 with another adult. The respondents typically (63.1%, n = 2055) brought chickens into their care at around 18 months of age (i.e., when they would otherwise be sent to slaughter at a typical egg farm). Over 24% (24.1%, n = 496) brought chickens into their care at sexual maturity (point of lay), whilst almost 6% (5.9%, n = 121) brought chickens into their care as chicks and 5.4% (n = 111) hatched chicks themselves. Only 1.5% (n = 31) brought chickens into their care at the age of two or older. 

**Figure 2:**
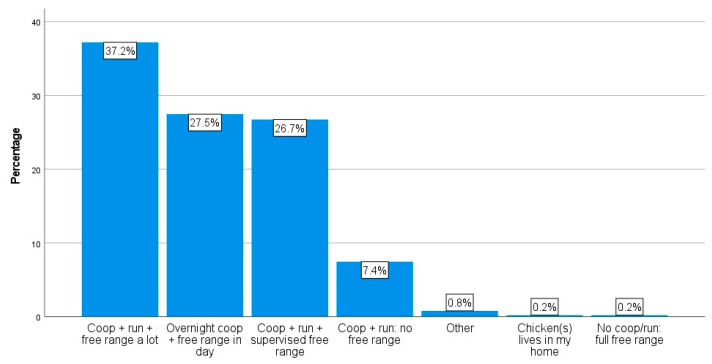
Respondents’ setup for their chickens’ living quarters are shown (n = 2054). Note: Respondents were provided with the following information: For the purposes of this questionnaire, “coop” refers to an indoor henhouse where chickens often sleep and “run” refers to an enclosed outdoor area in which chickens can move around in the open air.

As displayed in Figure 2, the home setup types for respondents’ chickens were quite variable; “a coop and run with free-range access a lot of the time” was the most common answer selected, although this option was only selected by 37.2% (n = 764) of the respondents. Fear of predation on chickens, selected by 61.2% of the respondents (n = 93), was the most common reason for not allowing any free-range access at all. Other reasons included the following: to prevent damage to the garden (34.9%, n = 53), not wishing their chickens to become too habituated to having regular garden access (9.2%, n = 14), and “other” (32.9%, n = 50). Common elaborations on “other” included, for example, escape risk, harm from dogs, concern over grass-eating impacting crops, and restricting harm to amphibians and invertebrates in their gardens.

Amongst the respondents who utilized a coop (n = 2048), approximately half (51.0%, n = 1045) removed feces daily, and 18.3% (n = 375) did so every few days. Around one quarter (24.1%, n = 493) removed feces weekly, and 3.5% removed feces monthly (n = 72). Only two respondents (0.1%) stated they never removed feces, and 2.0% (n = 40) used the “deep litter method”. Amongst the respondents with a run, 53.3% (n = 780) did not remove feces from the run on a daily basis, whilst 46.7% (n = 683) did. Most respondents (62.0%, n = 1257) had a wooden coop, whilst 33.4% (n = 677) had a plastic coop; 4.5% (n = 92) selected “other”. Just over a quarter of the respondents (25.8%, n = 526) used an automatic door opener on the coop or run, whilst nearly three-quarters did not (74.2%, n = 1516).

**Figure 3:**
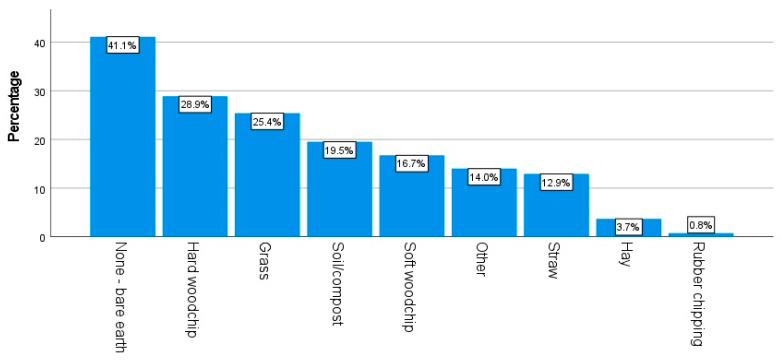
The run substrates used by respondents are shown. Note: Each answer choice was treated as its own independent dichotomous question (“yes” = substrate is used; “no” = substrate is not used); the percentages shown represent the percentage of respondents choosing “yes” for each choice of answer. Respondents could select multiple substrates, meaning the total number of respondents is not stated. This question was just for those who stated they used a run. NB: soft woodchips and bark are not recommended for use as a substrate for chickens [52].

**Figure 4:**
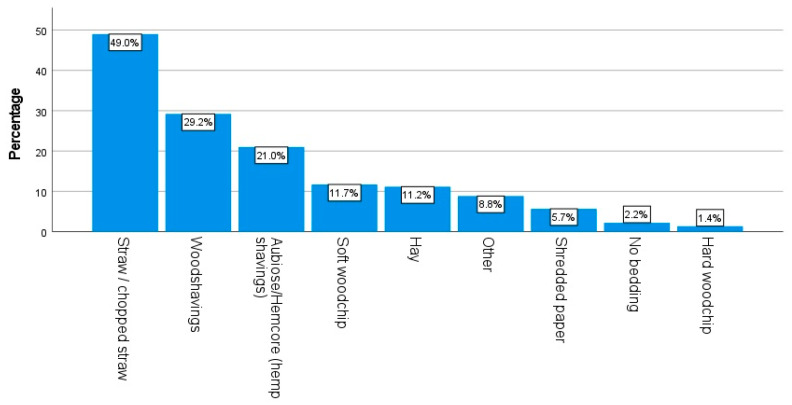
The coop bedding types used by respondents are shown. Note: Each choice of answer was treated as its own independent dichotomous question (“yes” = bedding is used; “no” = bedding is not used); the percentages shown represent the percentage of respondents choosing “yes” for each answer choice. Respondents could select multiple answer choices, meaning the total number of respondents is not stated. This question was just for those who stated they used a coop. NB: soft woodchips and bark are not recommended for use as any substrate for chickens [52].

**Figure 5:**
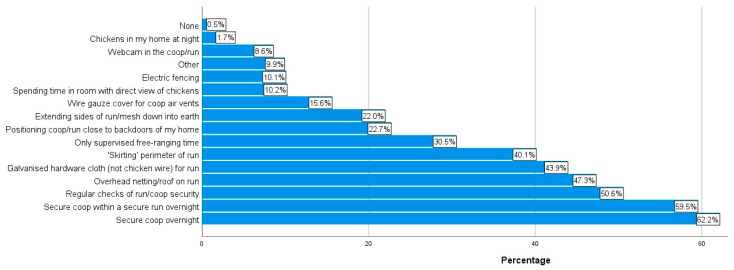
The steps taken by respondents to minimize predation risk are shown. Note: Each answer choice was treated as its own independent dichotomous question (“yes” = step was taken; “no” = step was not taken); the percentages shown represent the percentage of respondents choosing “yes” for each answer choice. Respondents could select multiple answer choices, meaning the total number of respondents is not stated. Common elaborations on “other” included, for example, using a galvanized mesh floor base, using security lights, having dogs/goats/alpacas, providing hiding places, and placing the run on a concrete base. Other responses included the use of CCTV (closed-circuit television), “human urine around run perimeter”, and flags. It was also common for respondents to have stated that their garden was “fully protected” and “nothing could get in”.

In terms of the size of run used by respondents, “2 m × 2 m or greater but smaller than 4 m × 4 m” and “4 m × 4 m or greater but smaller than 6 m × 6 m” were the most common options selected by 26.0% (n = 381) and 26.8% (n = 392) of the applicable respondents, respectively. Figure 3 and Figure 4 demonstrate the diverse array of run substrates and coop bedding used by the respondents. The most popular choice of run substrate was “bare earth” (41.1%, n = 602); the most popular choice of coop bedding was straw (49.0%, n = 991). Figure 5 displays the most and least common steps taken by respondents to minimize the risk of predation on their chickens. The most common minimization step practiced by the respondents was “keeping chickens in a secure coop overnight” (62.2%, n = 1278).

Regarding the bird flu restrictions in place in some countries in the winter of 2020–2021, 69.2% (n = 1420) stated they had followed the legal requirements to keep their chickens inaccessible to wild birds, 15.8% (n = 325) stated that they “partially” followed the requirements, and 4.0% (n = 83) stated that they did not follow the requirements. A further 8.0% of the respondents (n = 165) stated that there had not been any “chicken lockdown” in their country/region, and 2.9% (n = 60) selected “other”. Of the 85.0% of the respondents that followed the legal requirements or partially did so, 53.9% (n = 938) were worried about the welfare of their chickens during this “chicken lockdown”, and 17.1% (n = 297) “sometimes worried” about their welfare. 

#### 3.3.2. Health

##### Minimum Temperatures and Chicken Weights

The respondents most commonly (41.2%, n = 836) selected “−5 °C/23 °F” as the lowest temperature that chickens can cope well with on an ongoing basis. This was followed by “0 °C/32 °F” (27.9%, n = 565) and “5 °C/41 °F” (16.1%, n = 327). A further 14.8% (n = 300) selected “−10 °C/14 °F” or lower. The vast majority of respondents (57.8%, n = 1184) did not know the weight of their chickens; another 18.4% (n = 378) of respondents stated the weight of their chickens to be around 2 kg, 11.2% (n = 229) stated that the weight of their chickens was around 1.5 kg, and 8.2% (n = 169) stated the weight to be around 2.5 kg. Only 1.6% (n = 33) stated that the chickens were around 3 kg. Most people selecting “other” (2.7%, n = 56) stated they either had bantams weighing under 1 kg or large breeds weighing around 4 kg. 

##### Treatment and Veterinary Care

Over 63% of the respondents (63.9%, n = 1282) took their chickens to a veterinarian when ill; of these, 33.0% (n = 423) used a veterinarian specializing in exotic animals (an ‘exotics vet’). Of the 36.1% (n = 727) who did not use vets, 74% (n = 538) attempted to treat ill chickens at home, whilst the remaining 26.0% (n = 189) of those who did not use vets did not treat ill chickens at all. When the 538 respondents were asked whether their home treatment had proved successful, the vast majority (71.7%, n = 383) answered “Yes”. Only 2.8% (n = 15) answered “No”, with the remaining 25.5% (n = 136), answering “Partially”. When asked why they treated chickens at home rather than taking their chickens to the vet, the top reason, selected by 55.1% (n = 296), was “I fear vets will not know how to treat my chicken and/or will just suggest euthanasia”. This was followed by “Vets are too expensive”, which was selected by 45.3% of the respondents (n = 243), and “I believe in herbal remedies”, which was selected by 26.4% of the respondents (n = 142).

For those not using a vet specialized in exotic animals (42.8%, n = 859), the most commonly selected reason was the presence of an avian/chicken-friendly vet at a standard veterinary practice (47.7%, n = 407). Not knowing an exotic vet was the next most common reason (selected by 38.1%, n = 325), followed by an exotic vet being too far away (14.5%, n = 124) and being unsure what an exotic vet was (10.7%, n = 91). Exotic vets being too expensive was only chosen by 2.7% of the respondents (n = 23). Of those using any kind of vet, 73.6% (n = 939) were happy with the health and welfare services their chickens receive, 18.9% (n = 241) were unsure, and 7.5% (n = 96) were unhappy. Of the 96 respondents stating that they were unhappy, the main reasons cited in the 95 explanations provided were a lack of chicken expertise (despite supposedly being a “chicken friendly vet”), an exotic vet not being sympathetic to chickens, a vet being too eager to euthanize, exotic vets not necessarily being avian specialists, high costs, treatments not working, negative judgements, and many vets not being willing to offer the Suprelorin^®^ implant.

The vast majority of respondents (98.4%, n = 2018) continued to care for their chickens if they ceased laying eggs, with all other courses of action (namely, “I rehome her”, “I kill/have her killed”, “all hens are hormonally implanted to cease egg-laying”, and “I never have hens in my care”) being individually under 1%. Additionally, the respondents had mixed practices regarding vaccinating chickens (58.1% selected that they did/the farmers did, and 41.9% selected that they did not). 

##### Implanting Hens

Nearly 70% of the respondents (68.0%, n = 1393) had not heard of the Suprelorin^®^ hormonal implant for hens, whilst 32.0% (n = 655) had heard of it. Of those who had not heard of the implant, after reading some basic information about it (see Supplementary Materials A, Q29), 53.9% (n = 743) would still not consider using it. An additional 28.2% (n = 388) felt unsure, whilst 17.9% (n = 242) stated they would consider using it, although 13.9% (n = 192) of this 17.9% would require significant reductions in the cost of the implant. Qualitative (free text) explanations from 685 of those who remained unwilling to consider the implant in the future mainly focused on prohibitive costs, a perception of implants being too much of an intervention/unnatural/cruel, a preference for eggs, the perception that the hens were happy enough, and concerns about side effects.

The respondents who had heard of the implant for hens were asked if they thought it prolonged hens’ lives and increased the quality of hens’ lives. The respondents were divided roughly equally in opinion in each case between “Yes” (44.9% and 51.6%, respectively) and “Unsure” (53.4% and 44.6%, respectively), with only 1.7% and 3.8% saying “No”, respectively. However, 76.1% (n = 497) stated that they did not use it, whilst 19.6% (n = 128) used it either in a treatment (17.5%) or preventative capacity (2.1%). The remaining 4.3% (n = 28) selected either “other” or indicated they only cared for roosters. Using the implant in a treatment capacity meant that the hens were only implanted if health problems connected to egg-laying arose, whilst using the implant in a preventative capacity meant that the hens were routinely implanted to try and prevent health problems arising.

When those who had heard of the implants were asked if they had any concerns about them, 297 respondents answered in some detail. The main concerns cited were as follows: the initial side effects in terms of personality changes and hard molt (though it was emphasized these wore off with subsequent implants), costs (especially if homing many hens), occasionally unreliable function, short duration, some vets advising against implant use, chickens needing to be fit and well when receiving it, lack of access to the implants, more research on and knowledge of implants first being required, hens’ own hormones overriding the implant in Spring, the implant being unlicensed for chickens, lack of clarity regarding egg withdrawal times if only using the implant temporarily, flock dynamics if only using the implant on one or two hens, and knock-on health concerns (e.g., liver problems).

##### Chicken Deaths

**Figure 6:**
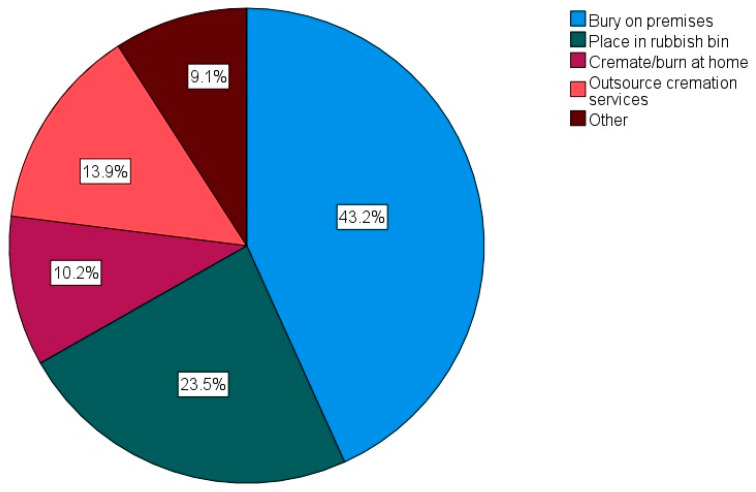
How respondents (n = 1878) tended to their chickens’ bodies after death is shown. Note: A common ‘other’ method was to bury or leave the bodies in a nearby field or wood so that other wild animals could make use of the corpse.

The vast majority of the respondents (93.6%, n = 1923) did/would not kill their chickens for human consumption. Of the 6.4% (n = 131) who did/would kill their chickens, the main method used was cervical dislocation (57.3%, n = 75), with roughly an equal proportion of the remaining respondents selecting either the use of a killing cone (chicken is restrained upside down with the head extending through the narrow end of a cone-shaped device, allowing the neck to be clamped for dislocation), decapitation, a mix of methods, or “other”. Figure 6 displays how the respondents tended to their chickens’ bodies; the most popular method was to bury the bodies at home (43.2%, n = 812). This is often an illegal activity in many countries due to the classification of chickens as a food species and concerns relating to the spreading of diseases [53]. The majority of the respondents (62.0%, n = 1223) did not memorialize their chickens; however, 25.3% (n = 500) did, and a further 12.7% (n = 250) sometimes did. Further information regarding chicken deaths and other aspects of health and welfare are reported in Section 3.8.

#### 3.3.3. Other Care-Taking Practices

Table 1 displays the results of the series of Likert-type items concerning the frequency of various chicken care-taking practices. The respondents’ frequencies of desirable care-taking practices were generally quite variable, with the means for each item on the Care Series ranging from between 1.35 (never–rarely) and 4.61 (often–always), and only five items were rated above 4. Cronbach’s alpha was 0.73, suggesting acceptable internal consistency. The standard deviations are also shown for each series item; these were under one for six items and never exceeded 1.56. 

### 3.4. What Attitudes towards Chickens Do Chicken Carers Have? 

Table 2 displays the results of the CAS. Generally, the respondents’ attitudes towards chickens were quite variable, with the means for each item on the CAS ranging from agreement levels of 1.9 to 4.88. Cronbach’s alpha was 0.74, suggesting high internal consistency. The standard deviations were lower than one for four items (1, 3, 5, and 7), and never exceeded 1.34. The most popular role name was “chicken keeper” (37.1%, n = 761), followed closely by “chicken carer” (36.6%, n = 751). “Chicken rescuer” was selected by 21.2% (n = 435), whilst “backyard farmer” was selected by 3.1% (n = 63). Common elaborations upon “other” (selected by 2.0%, n = 42) included “chicken mum”, “companion”, “friend”, “pet owner”, “chicken owner”, and “chicken dad”. Over three-quarters (76.4%, n = 1566) of respondents named their chickens, with 7.2% (n = 148) stating they did not; the remaining 16.4% (n = 336) sometimes named their chickens. In preparation for chicken care if the owner were to die, 53.5% (n = 1097) had some plans in place, and 46.5% did not (n = 954). Almost three-quarters of respondents (72.7%, n = 1494) thought they would still be caring for chickens in 10 years’ time; 4.5% (n = 93) stated they would not be, and 22.7% (n = 467) were unsure (data regarding the qualitative explanations are available upon request). 

### 3.5. What Type of Relationship Do Chicken Carers Have with Their Chickens?

Table 3 displays the results of the adapted OBRS. The item with the highest level of agreement amongst the respondents was “When my chicken is ill, it is my duty to care for him/her” (98.8% agree, n = 2026). When answering the OBRS questions, 63.8% felt able to select a chicken to whom they felt closest. The PCA with varimax rotation revealed that four components explain 60.9% of the variance at an Eigenvalue of 1, meaning there were four dimensions to the owner–chicken relationship. The original labels for each dimension provided by Burmeister et al. [34] were adopted in this study: “bird as human”; “social support”; “empathy, attentiveness, and respect” (hereafter, shortened to “empathy”); and “relationship of bird toward the owner” (hereafter shortened to bird reciprocity). Table 4 demonstrates how the items were mapped onto each dimension. The first three dimensions had a Cronbach’s alpha of over 0.8 (considered very good); the fourth dimension’s Cronbach’s alpha was 0.67 (considered acceptable). Thus, the subscales, as well as the overall scale (with a Cronbach’s alpha of 0.914), demonstrated very good internal consistency. Moreover, the Kaiser–Meyer–Olkin Measure of Sampling Adequacy was 0.931, suggesting a very compact pattern of correlations and, hence, reliable dimensions.

Table 4 also displays the mean scores and standard deviations across all respondents for each item of the OBRS. Generally, the respondents had a “personal” relationship (versus “impersonal” at the lower end of the spectrum or “close personal” at the upper end of the spectrum) with their chickens. Respondents scored highest on the third dimension (Empathy); the means are all above 4.4, and the standard deviations are all below 0.9, indicating the high levels of empathy that respondents have towards their chickens.

### 3.6. Significant Differences in Care, Relationship, and Attitude between Different Types of Carer

From the 264 chi-squared associations tested (including the likelihood ratio test where applicable) between care-taking and demographic variables, 156 associations were deemed to be significant (59.1% of all associations tested), and 108 non-significant associations (40.9% of all associations tested) were found. Cramer’s V value never reached above 0.272. The full significance and Cramer’s V results can be seen in Table S2 in Supplementary Materials C. Cramer’s V was 0.2 or more in seven cases (i.e., on the high end of a low/weak strength association). These cases were as follows:

- Where respondents source chickens and country of residence;
- Whether respondents care for hens/cocks/both and country of residence;
- Where respondents sourced most of their chickens and preferred role name;
- Whether respondents regularly use implants and gender;
- Whether respondents regularly use implants and diets;
- Whether respondents had heard of the implants and human age; and
- Whether respondents had heard of the implants and diet.

The patterns of these seven cases will be introduced briefly in turn; full details can be found in Tables S3–S9 in Supplementary Materials C. In terms of where the respondents sourced their chickens and their country of residence (LR = 188.885, df = 42, n = 2045, *p* < 0.001, V = 0.203), 62.5% of British residents selected ex-commercial chickens in contrast to only 25.0% of EU residents and 13.0% of American respondents. Considering the source of the chickens and how they named their role (LR = 450.917, df = 24, n = 2052, *p* < 0.001, V = 0.214), 60.9% of those choosing the role name “chicken carer” primarily had ex-commercial chickens in their care, in contrast to 26.0% with the same role name having chickens from a local breeder. Regarding whether respondents have hens/cocks/both and their country of residence (LR = 68.467, df = 14, n = 2036, *p* < 0.001, V = 0.272), over 44% more American residents cared for both hens and cocks than residents in the UK (62.2% and 17.6%, respectively). 

Regarding regular implant use and gender (LR = 26.616, df = 12, n = 653, *p* = 0.009, V = 0.227), 40.0% of males used implants to some extent versus just 18.3% of females (among those who had previously heard of the implant). In relation to regular implant use and diet, it is most useful to consider the “diet group”, (versus the ungrouped diet variable), which still yielded significant results, albeit with a Cramer’s V just under the 0.2 level (LR = 47.291, df = 12, n = 652, *p* < 0.001, V = 0.198): 36.5% of vegan/plant-based carers used the implant in either a preventative or treatment capacity versus 21.4% of vegetarians and 16.6% of omnivores.

Regarding previous knowledge of the implants and human age group (X^2^ = 90.816, df = 6, n = 2044, *p* < 0.001, V = 0.211), 18.3% of the 65–74 age group, 27.9% of the 55–64 age group, and 33.5% of the 45–54 age group had heard of the Suprelorin^®^ implant; this is relative to 54.5% of the 25–34 age group and 42.1% of the 35–44 age group. Regarding prior knowledge of the implant and diet group (X^2^ = 73.384, df = 3, n = 2042, *p* < 0.001, V = 0.190), the percentage of respondents aware of the implant prior to this survey increased the more the respondents abstained from consuming animal products: 27.9%, 40.4%, and 64.9% selected awareness of the implant for omnivores, vegetarians, and vegan/plant-based, respectively.

Table 5 details significant differences in the mean scores of the respondents across the Care Series, CAS, and OBRS between different groups (i.e., types/style of carer). From 308 tests of differences between the groups (using either the Kruskal–Wallis or Mann–Whitney U test as detailed in Table 5), 226 (73.4%) were deemed significant, and 82 (26.6%) were deemed non-significant. The following exemplar variables, diet, diet group, whether respondents would consider implant use, where respondents sourced most of their chickens, country of residence, number of chickens, whether respondents name chickens, and vet use, were examined more closely using the Bonferroni post hoc test. This demonstrated the specific groups that generated the significant differences, as per Table 6. 

### 3.7. Is There a Relationship between the Care-Taking Practices, the Human–Chicken Relationship, or Attitudes towards Chickens?

Table 7 highlights Spearman’s rho correlation coefficients existing between the CAS and individual Care Series items and between the OBRS and individual Care Series items. Of 150 possible correlations, 41 had moderate positive correlations. Table 8 demonstrates weak–strong positive associations between the respondents’ CAS and OBRS scores and Care Series and OBRS scores, with Spearman’s rho correlation coefficients ranging from 0.176 to 0.543. Spearman’s rho further demonstrated a moderate positive relationship between respondents’ CAS and Care Series scores (0.351, *p* < 0.001).

### 3.8. What Are the Biggest Challenges Owners Experience in Safeguarding Chicken Welfare?

**Figure 7:**
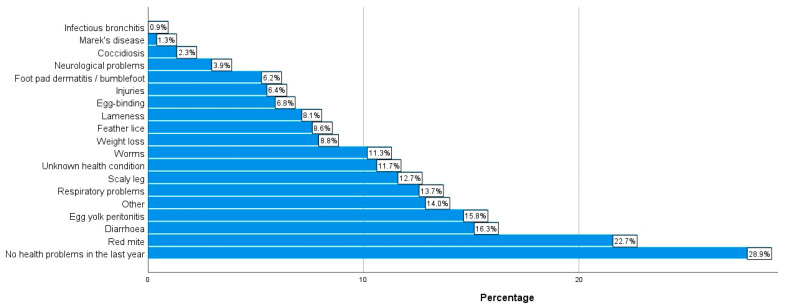
Health conditions experienced in respondents’ chickens within the last year are shown. Note: Each answer choice was treated as its own independent dichotomous question (“yes” = health condition was experienced; “no” = health condition was not experienced); the percentages shown represent the percentage of respondents choosing “yes” for each answer choice. Respondents could select multiple answer choices, meaning the total number of respondents is not stated. “Other” conditions include, for example, crop problems (sour crop/impacted crop/pendulous crop), fowl pox, lash egg, tumors, eye problems, flystrike, water belly, vent gleet, mycoplasma, and prolapses. NB: Respondents’ level of expertise was not evaluated; however, for those using a vet, knowledge of health conditions would be informed by veterinary diagnoses.

**Figure 8:**
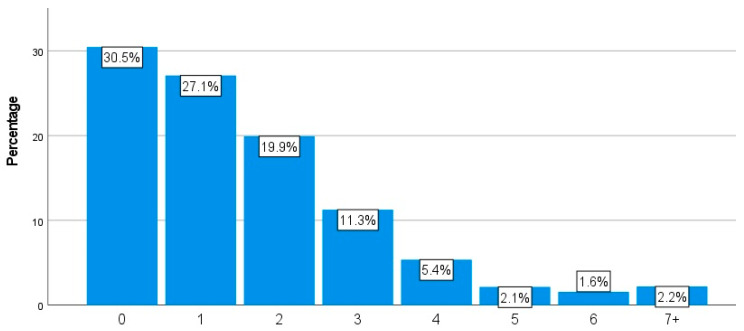
Number of respondents’ chickens who had died within the last year (n = 2052).

**Figure 9:**
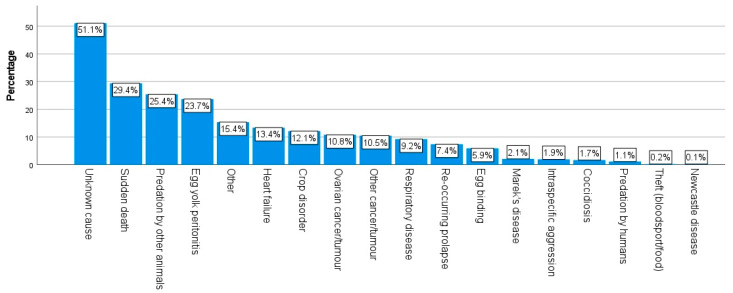
Causes of death in respondents’ chickens who had died within the last year are shown. Note: Each answer choice was treated as its own independent dichotomous question (“yes” = cause of death was applicable; “no” = cause of death was not applicable); the percentages shown represent the percentage of respondents choosing “yes” for each answer choice. Respondents could select multiple answer choices, meaning the total number of respondents is not stated. Elaborations on “other” showed that many selected this option if they had not experienced any chicken deaths yet. Other relevant comments included traffic accidents, neurological conditions, drowning, ascites, fatty liver disease, salpingitis, flystrike, canker, breaking of the neck in a fence, and abscesses. NB: Respondents’ level of expertise was not evaluated; however, for those using a vet, knowledge of the cause of death could arise from veterinary diagnoses.

As can be seen in Figure 7, 71.1% of the respondents (n = 589) reported some kind of health condition in their chickens within the last year. Topping the list of health conditions selected were red mite (22.7%, n = 462), diarrhea (16.3%, n = 331), and EYP (15.8%, n = 321). When considering the number of chickens who had died in the last year, approximately 30% (30.5%, n = 625) stated that none of their chickens had died in the last year. In contrast, nearly 70% (69.5%, n = 1427) stated they had experienced the death of some of their chickens in the last year; this ranged from one to more than seven chicken deaths, as per the information in Figure 8. Figure 9 details the most common causes of death amongst the respondents’ chickens. The most commonly selected option was “Unknown cause” (51.1%, n = 951). 

**Figure 10:**
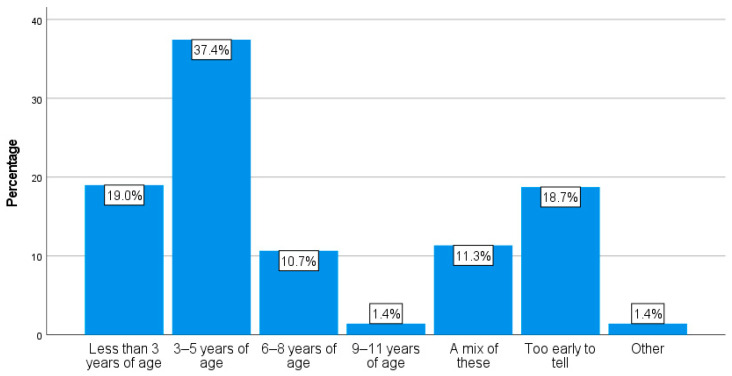
The average age that respondents’ chickens lived to (n = 2054) is shown. Note: Elaborations on “other” for this question included older than 11 years, unknown ages, and none having died yet.

**Figure 11:**
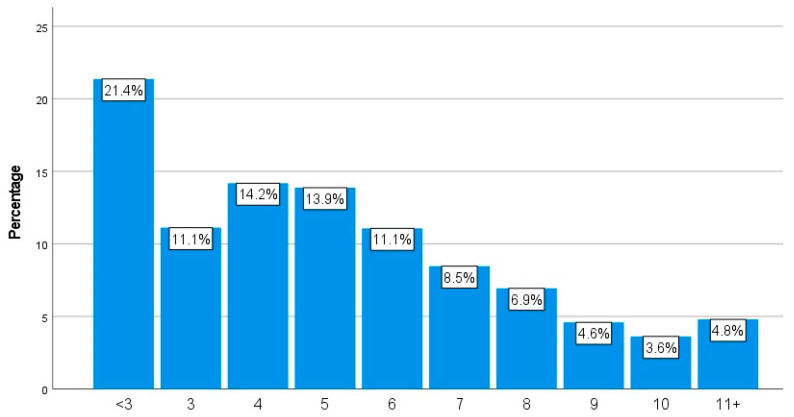
The ages of respondents’ longest-living chicken so far (n = 1961) are shown. Note: Elaborations on the “other” answer option revealed one chicken living to the age of 17. Respondents also stated that ex-commercial hens did not live as long as pure breeds.

Figure 10 displays the considerable variation in the average age of the respondents’ chickens at their time of death. For instance, almost 20% (19.0%, n = 390) stated that, on average, their chickens died younger than three years of age; however, 12.1% (n = 248) selected ages between six and eleven. In terms of the oldest age reached, Figure 11 details how, for example, 21.4% (n = 419) of the respondents had not yet had a chicken who reached three years of age, whilst 4.8% (n = 94) had a chicken older than eleven. 

Nearly 90% (87.8%, n = 1800 respondents) stated no presence of abnormal behavior amongst their chickens, with 250 (12.2%) stating the presence of abnormal behavior. The respondents (n = 248) who stated the presence of abnormal behaviors included behaviors such as excessive feather pecking (especially during bird flu restrictions), feather eating, pacing the perimeter of the run, self feather plucking, sudden jumping fits, extended broodiness, excessive mounting, hens displaying rooster behavior, and stalking each other. Whether these aforementioned behaviors all qualify as abnormal behavior is discussed in Supplementary Materials D. The vast majority of respondents (92.6%, n = 1901) reported that their chickens had mostly full plumage; a further 6.7% (n = 138) stated that their chickens “sometimes” had full plumage. Moreover, 24.0% of the participants responded either positively or with “sometimes” to the statement that their chickens’ hind feathers were regularly soiled to the extent that it was hard to manage. 

Based on 1644 qualitative responses to the question about how their chickens’ welfare could be further optimized, excluding the respondents who reported that their chickens’ welfare levels were already high enough (n = 209), the top three subcategories were “more space” (n = 511), “effective predator protection” (n = 193), and “better vet care” (n = 109). Based on 1362 qualitative responses to the question about what improved support could help enhance their welfare, excluding the respondents not wanting to provide further help (n = 192), the top three subcategories were “knowledgeable avian vets available locally” (n = 501), local specialists/experienced carers (n = 94), and “cheaper vets” (n = 85). Further details on these qualitative responses, in addition to general comments and other novel care-taking practices cited, can be found in Tables S10–S13 in Supplementary Materials C. It should be noted that respondents’ expertise levels were not evaluated.

## 4. Discussion

This chapter focuses on the most novel results arising from this study. The authors recommend viewing Supplementary Materials D for a discussion of the results that supplement the existing literature and span demographics; keeping chickens as pets; veterinary care for chickens; and other welfare considerations, including higher reported death and illness rates, welfare impacts arising from avian flu lockdowns, potentially inadequate chicken living spaces, and the methods used to kill chickens (for the 6.4% who do kill their chickens for consumption). The authors also encourage scrutiny of Section 3 for full consideration of all the results from this study. Recommendations for future study are integrated throughout this discussion, and the chapter ends with an acknowledgement of the study’s limitations. 

### 4.1. Implants

For the first time (to the authors’ knowledge), empirical data on the use of Suprelorin^®^ hormonal implants by chicken owners were collected in this study. In addition to 68.0% of respondents not having heard of this treatment option (hereafter named implant naive), over three-quarters (76.1%) of those who had heard of it did not use it (hereafter named implant aware). A further 19.6% did use it. In a treatment capacity, 17.5% used it; and in a prophylactic capacity, 2.1% used it. This equates to just 6.3% of the entire questionnaire sample. Thus, it is clear that the use of this implant remains quite a niche practice, though it is potentially growing, as 6.3% is still a significant number; for instance, it is a higher percentage than the number of vegans in this study (4.7%), so it is also clear that it is not only vegan carers utilizing this treatment. Additionally, when the implant naive were presented with a brief summary of its purpose, cost, and implementation (see Supplementary Materials A, Q29), there was roughly a 50:50 split amongst those who stated they would still not consider it and those who might or would consider it. This suggests that more hen owners (and hens) could benefit from owners hearing about this treatment option. 

The main concerns cited about the implants by the implant naive were prohibitive costs, implants being too much of an intervention, a preference for eggs, hens already being happy enough, and concern about side effects. Amongst the implant aware, the same concerns (apart from thinking hens were happy enough) were raised in addition to occasional malfunctioning implants, short duration of the implant, access issues, wanting more research on implants prior to use, some vets advising against use, hens needing to be sufficiently well, lack of licensing for chickens, possible knock-on health effects, and impacts on flock dynamics if only a couple of hens are on the implants. This longer list of concerns is expected due to the prior knowledge and, for some, the experience of the implants. These findings demonstrate how more consistent information regarding the implants is required among veterinarians. These findings also suggest more awareness is required about the benefits of using implants prophylactically rather than just in a treatment capacity. Methods to decrease the costs of these implants would be hugely welcomed and allow greater uptake by chicken owners, as would more research into their long-term use. Such research could include gathering the views of different stakeholders (such as vets, rehoming charities, and former chicken carers) to prevent biases and enable corroboration in terms of the findings. The use of complementary methodologies, such as the mining of veterinary records and comparative behavioral analyses of chickens kept under different conditions/treatments (such as with and without the implant), would further corroborate (or challenge) the results of this study.

Despite chicken caring being a female-dominant endeavor and the vast majority of respondents to this survey being female (86.9%), there were significant differences between males and females regarding regular implant use, with males being more than twice as likely to use the implants than females. Vegans/plant-based dieters were also found to be more likely than vegetarians and omnivores (over one-and-a-half times and twice as likely, respectively) to both have heard of the implants and utilize implants. This could mean that the implants are promoted more in vegan than nonvegan circles. As vegans do not want their hens’ eggs for consumption, this may facilitate consideration of implant use as vegans do not believe egg consumption to be “natural, normal, and necessary”, which is described as the 3N justifications for egg consumption by nonvegans [54]. Studies also suggest that vegans may be more likely to have more detailed knowledge about some farming practices, such as the harm of selectively breeding for maximum egg production (e.g., see [55]). Younger respondents were also more likely to have heard of the implants than older respondents. This mirrors the phenomenon of greater open-mindedness amongst younger age groups [56]. The result of more males using the implant than females should be corroborated and investigated in further studies.

Another complication is that there is a discrepancy between different countries regarding the official legal status of using these implants in hens. For instance, despite chickens being solely classified as “food animals” in both the UK and the USA, the implant’s extra-label use in chickens is legal in the UK (whilst officially licensed only for ferrets and dogs); however, it is illegal in the USA, where its extra-label use is not permitted by the FDA [23,57]. Nevertheless, there is evidence of its considerable use (based on private communications of chicken owners based in the USA with the author), which is a risk for American veterinarians to take, and there are efforts to change this, as evidenced by petitions [58]. This links to the qualitative comments provided in this study about the need to change the legal status of household chickens away from livestock and towards pets and to properly license medications for chickens rather than simply relying on the cascade method, which allows for the use of unlicensed products in certain circumstances [53]. Finally, current evidence/advice suggests prophylactic use is best [23]; however, amongst the few respondents who used the implants, most only used them in a treatment capacity once problems arose.

### 4.2. Diet/Consumption Habits of Chicken Carers

For the first time in a quantitative survey, chicken owners were asked about their diet category. Vegetarians and vegans were of higher proportions (17.6% and 4.7%, respectively) than within the general population, for instance, if considering the vegetarian and vegan proportions of the general populations in the UK (5.0% and 2.0%, respectively; [59]) and US (5.0% and 4.0%, respectively; [60]). As the majority of participants in this study were from the UK, it is particularly notable that the proportions of vegetarians and vegans were more than double those of the general UK population. This could be due to the purposive sampling methods used. However, vegetarians and vegans have been found in higher proportions amongst animal carers/pet owners than in the general population (e.g., see [61]). Moreover, given the high number of ex-commercial hen carers amongst this study’s respondents, one might expect a higher number of vegetarians and vegans, as they are actively intervening in the business-as-usual route to slaughter for commercial chickens. 

Those who are omnivores and only refrain from consuming chicken meat amounted to 2.0%. It seems that whilst chicken carers, on the whole, see their own chickens as inedible, they still continue to eat other chickens. Macauley [30] (p. 28) suggests that this could be due to the meat-animal disconnect in that chicken carers do not associate the chicken meat they purchase and eat with actual chickens. Moreover, it could be due to an “ethic of proximity” ([62] (p. 162), [63]), in that not killing and eating their own chickens seems immediately within their carers’ power whilst battling against the cultural norm of eating meat and against industrialized animal farming may seem less surmountable to an individual and less morally urgent as the harm to animals is more hidden and distant. 

There was a high–weak positive association between all scales/series in this study (i.e., the CAS, OBRS, and Care Series) and diet group, insofar as the more respondents abstained from consuming animals or their derivatives, the higher they scored on each of the scales/series. This could suggest that respondents’ meat, dairy, and egg consumption are potentially not just an inconsequential attitudinal/behavioral inconsistency in relation to their chickens but that greater abstinence from consuming animals or their derivatives could lead to improved human–chicken relationships, more positive attitudes towards chickens, and improved care-taking practices. This warrants further research.

### 4.3. Indoor/Disabled Chickens, Hens Eating Eggs, Chicken Sweaters, and Chicken Rescue

For the first time (to the authors’ knowledge), in this study, chicken owners were surveyed about any practice relating to keeping chickens predominantly indoors, caring for disabled chickens, and feeding hens’ eggs back to them. This study found that 12.1% of the respondents had cared for a (predominantly) indoor chicken, and 19.7% of respondents had cared for a chicken with special needs. Additionally, 21.6% of the respondents regularly let their chickens into their home—18.1% for long periods. This could potentially indicate that chicken caring may be beginning to mirror dogs’ journey from backyards (workers) to living rooms and even beds (companion animals), with a similar shift in attitude from dirty to cute and from minimal to significant spending on healthcare. In the present study, 65.6% responded “sometimes”, ‘often”, or “always” to the statement, “Regardless of the cost, if there is a treatment that could help my chicken, then I pay for it or fundraise for it if I cannot afford it”. 

There are, however, some strong opponents to keeping chickens indoors for long periods of time, including veterinarians (e.g., see [64] (pp. 401–402)). The welfare concerns seem to mainly center around possible deprivation in terms of highly motivated behaviors such as dustbathing and ensuring that extra husbandry needs would be fulfilled, such as nail clipping, ingestion of household objects, and unsuitably warm temperatures. It is conceivable that all needs could be met by the most committed of carers though, and some claim there are also notable welfare benefits for chickens when sharing homes with humans, including predator protection and protection from extreme weather conditions, for instance, see [65]. In terms of disabled chickens, there are concerns over when to judge their quality of life as too severely impaired for it to constitute a “life worth living” [66]. The development of quality-of-life scales specifically for chickens—comparable to those existing for dogs and cats—would aid in these decisions.

Moreover, 32.4% of the respondents stated that they feed their hens’ eggs back to them (8.9%, “regularly”/“always”; 23.5%, “sometimes”). This is a significant number when considering that backyard and commercial chicken keeping is renowned for going to grave efforts to prevent chickens from eating their own eggs; some agricultural scholars even describe it as a behavioral disorder or disease (e.g., [67] (p. 46), [68]) despite the phenomenon of placentophagy in mammals and it simply being a result of positive reinforcement in relation to the nice taste if chickens should ever come across a broken egg [69] (p. 451). In future research, it would be interesting to investigate whether this is a growing practice and to acquire some clinical research regarding the possible health implications of this practice in the long term for both chickens’ consumption of the shell and the contents. There are strong beliefs concerning the health benefits by proponents of the practice as well as increasing recognition amongst backyard chicken keepers about the potential benefits of feeding shells back to chickens; however, there seems to be no clinical evidence for this yet, especially regarding the feeding of the shell’s contents (e.g., [70,71]). The theory is that in lieu of using implants in a preventative capacity, feeding hens’ eggs back to laying hens is the ‘next best thing’ to replace some of the nutrients lost during the creation of eggs.

An example of a novel care-taking practice that may cause welfare concerns is shown in the 8.4% of respondents who stated that they at least occasionally, if not more frequently, utilize chicken sweaters. Sweaters may protect against harsh temperatures when a chicken has few feathers; however, anecdotally, there is a concern that these may impede new feather growth and interfere with the self-regulation of a chicken’s temperature (e.g., see [72]).

With 56.1% of the respondents selecting “to prevent commercial chickens going to slaughter” as a primary motive for their chicken care, this study has quantitatively recognized a major new motive for caring for household chickens: preventing the slaughter of ex-commercial hens. The value chicken carers place on aiding ex-commercial hens in this way has been recognized previously (e.g., by Zoubek [17]); however, it has not been recognized as a major motive nor as a distinguishing feature between different types of carers that have been described by, for instance, Zoubek [17] or Souvestre et al. [18]. This reinforces the aforementioned (Section 1.2) subversive nature of at least a significant proportion of household chicken caring. It relates to notions of an “ethic of care” [20] (p. 90) and “entangled empathy” (as cited in [20] (p. 21)) and conveys an attitude that is “beyond eggs” [20] (p. 90).

### 4.4. Correlations with Chicken Relational Closeness: Anthropomorphism

Via the OBRS, this study found significant positive correlations between relational closeness and (1) owner attitudes in terms of the CAS (especially the third dimension of the OBRS [empathy]: moderate), (2) some individual Care Series items (moderate), and (3) the Care Series score (especially the first dimension of the OBRS [bird as human]: high moderate). Of particular interest is the latter point: a high moderate positive association between the Care Series score and the first OBRS dimension, the bird as a human, which describes the extent of anthropomorphism practiced by the owner. This is because it counters the result found by Burmeister et al. [73], who found a negative correlation between anthropomorphism and pet bird welfare. Specifically, the authors found significant correlations of the first and fourth dimensions of the OBRS (“bird as human” and “reciprocity from bird”, respectively) with bird behavior, which often indicates welfare problems (e.g., feather plucking, aggression towards humans/other pets, and locomotor stereotypies). However, crucially, they found “ornamental poultry” (including chickens) to be at greater risk of neglect or inattentiveness by their owners than anthropomorphism by their owners. 

To elaborate on a saying that the aforementioned authors used, “a dog is not a cat is not a bird”, it is also true that a parrot is not a finch is not a chicken, meaning more research is required to see if the findings of Burmeister et al. [73] can be replicated in relation to different types of pet birds. This is especially the case as, otherwise, to date, there is considerable research suggesting that anthropomorphic tendencies usually positively correlate with animal welfare, and this present study similarly suggested this is the case for chickens. Nevertheless, it is easy to understand that anthropomorphism may go too far and be detrimental to animal welfare. This could perhaps relate to this present study when considering the quality of life for disabled/special needs chickens; more research is required. It may also apply in terms of trying to fit a dog or cat mold onto pet chickens, whereby there is a conflation of human expectation/wants based on traditional pets with less traditional pet animals’ actual needs—“traditional-petamorphism” could be a potential name for this. Thus, conceivably, there could be a cut-off point for when anthropomorphic tendencies cease to be beneficial for pet welfare, including chicken welfare. To aid further research in this area, the development of a validated chicken care-taking scale would be beneficial.

### 4.5. Roosters

This project intended to draw attention to the care of roosters; however, only 0.1% (n = 3) of the respondents cared solely for roosters, whilst a further 19.1% (n = 390) cared for both roosters and hens. Regarding why people do not care for roosters, 63.9% stated it was because roosters are too noisy for their neighbors and 37.4% stated it was because they want eggs and are not interested in breeding. The third most commonly selected answer was that roosters were too noisy for the respondents (21.3%). In fourth place was not knowing enough about roosters (13.5%), followed by not being allowed to keep roosters due to their local council (12.5%) in fifth place, and finding roosters too aggressive (11.1%) in sixth place. A further 6.3% did not know it was an option to rehome roosters. Collectively, these results suggest that some information campaigns could enable more homes to become available for roosters. Information campaigns could target any additional knowledge required to care for roosters relative to hens and could counter their negative reputation as irreversibly aggressive, akin to the campaigns of Magnus and Griffler [74]. This is an urgent welfare need as there is a huge welfare problem regarding unwanted roosters, which is also well documented (e.g., see [22,75]). Research, regarding both relational aspects and welfare status, should be tailored to roosters in the future and for both rooster-only and mixed flocks.

### 4.6. Study Limitations

There were five main limitations of this study, as follows: First, there was substantial bias towards ex-commercial hens and the UK. This was, in large part, due to the number of BHWT supporters who responded to the charity’s request to complete the survey without an equivalent organization from other countries being used. However, such a well-established charity akin to the BHWT does not exist to the same extent in many other Western countries. Thus, it proved trickier to recruit participants from other countries. The questionnaire was also only made available in English, which further restricted participation from other countries. This means that the results are not necessarily generalizable to all non-commercial chicken carers or all countries. The findings similarly are not generalizable to all rooster carers due to the low number of respondents caring solely for roosters. Second, the questionnaire would have benefitted from more precision regarding a few more terms to avoid differences in interpretation, such as providing more objective descriptions of a “secure run” and “large” garden—the questionnaire did not state what counted as a secure run or large garden. Third, significant results may be underplayed as significant results may be even more significant than suggested by Cramer’s V [50]. Fourth, some key aspects were missed in the questionnaire; neither the level of welfare expertise nor knowledge regarding relevant avian flu legislation in terms of the respondents were evaluated, meaning that the results could not be contextualized in this regard. This may impair the reliability of some results; for instance, the number of abnormal behaviors and the extent of adherence to avian flu legislation may be over- or under-reported. Moreover, survey responses may not fully reflect the reality of a sensitive issue such as killing one’s own chickens, which should be taken into consideration when designing applicable questions. Fifth, due to the purposive sampling used when recruiting the participants, it should be noted that the proportions of different chicken owner types will likely not represent the general non-commercial chicken carer population, such as the number of vegetarian and vegan owners, as discussed in Section 4.2. Nevertheless, as a large-scale study based on over 2000 responses, there was a high degree of statistical reliability among this study’s results. Further research, as recommended in each of the previous subsections of this section, will further contextualize these present findings.

## 5. Conclusions

This questionnaire-based study surveyed 2000+ respondents. It focused on the care-taking practices of non-commercial chicken owners, these owners’ general attitudes towards chickens, and the owners’ human–chicken relationships. The study also investigated the possible correlations between these aspects and how chicken welfare might be further optimized. The study found variable care-taking practices but largely positive attitudes towards chickens and that respondents generally had a “personal” (though not “close personal”) relationship with their chickens, as defined by the Owner–Bird Relationship Scale. Additionally, care-taking scores, attitude scores, and relationship scores were found to be correlated.

Far-ranging data were collected; however, the most important results to underscore were as follows: First, the study reinforced previous findings of backyard chickens being viewed as pets (93.6% would never kill their chickens for consumption, 76.3% did not see chickens as morally less important than dogs, and 68.8% disagreed with breeding chickens for maximum egg production). Relatedly, 56.1% selected “preventing commercial chickens from going to slaughter” as their main reason for caring for chickens, thereby establishing the ex-commercial chicken carer as a new type of chicken carer not previously clearly identified. Compared to similar studies, this study found a much higher prevalence of reported health conditions and overall mortality. This is potentially connected to the high proportion of ex-commercial chicken carers in this study: 69.6% of the respondents. Similarly, for the first time, egg yolk peritonitis was identified as a leading health condition and cause of death. Other key welfare concerns reported include predation as a leading cause of death and concerns about chicken welfare during avian influenza restrictions that are common in many areas over winter (reported by 71.0%). 

For the first time in a questionnaire-based study, this study enquired about Suprelorin^®^ hormonal implant use to prevent egg laying and found that (1) 68.0% had not heard of the Suprelorin^®^ implant, and (2) only 6.3% used it at all: using it mainly in a treatment capacity. Additionally, for the first time, this study enquired about the dietary habits of chicken carers. Whilst this study found approximately double the proportion of vegetarians and vegans amongst non-commercial chicken owners compared to the general population (taking the UK as an example), the vast majority of chicken carers still follow an omnivorous diet that includes chicken meat and eggs. There were significant differences in respondents’ scores in terms of the Chicken Attitude Scale, the Owner–Bird Relationship Scale, and the Care Series, according to their diet group; the more animal products that were removed from the diet, the higher these scores became.

This study further found that 12.1% of the respondents had cared for predominantly indoor chickens, 19.7% had cared for chickens with special needs, and 32.4% practiced feeding hens’ eggs back to them to some degree. Roosters were present in the flocks of 19.1% of the respondents; however, only 0.1% cared solely for roosters. Finally, this study echoed findings from previous studies regarding the main demographics involved in non-commercial chicken care as well as the need for more avian-specialist, locally available, and affordable veterinary care for chickens. Due to the purposive sampling methods used, the results should be interpreted with caution. They may not reflect the broader population of non-commercial chicken carers.

As a consequence of this research, education campaigns are recommended concerning (1) the benefits of the Suprelorin^®^ implants for hens, (2) post-mortem services, and (3) rooster care. Campaigns are also warranted in response to shifting societal perceptions of chickens away from livestock and towards pets, especially in relation to non-commercial chicken status in the law and in licensing more medications specifically for chickens. Moreover, research is recommended into, inter alia, Suprelorin^®^ implant use, rooster-specific care, and further specific requirements in terms of caring for ex-commercial chickens.

## Figures and Tables

**Table 1:** Care Series: Respondents’ reported frequency of different care-taking practices, including mean frequencies and standard deviations. Note: 10 items were excluded from the final series as their impact on chicken welfare is debatable. The results for these items can be seen in Table S1 in Supplementary Materials C.

	Never	Rarely	Some-Times	Often	Always	Mean	Std. Deviation
I arrange for someone to care for my chickens when I am away for a weekend	3.9%	1.9%	5.0%	6.9%	82.3%	4.6144	0.95841
I ensure my chickens have fresh material for dustbathing in	1.2%	1.9%	8.2%	24.7%	64.0%	4.4799	0.81690
I try to improve my knowledge of optimal care-taking practices for chickens	0.9%	2.2%	13.0%	33.0%	50.9%	4.3088	0.83605
I spend time observing my chickens’ behavior	0.3%	1.3%	14.1%	45.5%	38.8%	4.2087	0.74941
I conduct periodical health checks of my chickens	1.1%	3.8%	19.8%	42.0%	33.4%	4.0238	0.88739
I introduce new chickens to a flock gradually over a period of a few weeks	12.2%	5.8%	13.5%	13.1%	55.4%	3.9507	1.40565
I clean my chickens’ hind feathers if they become very soiled	7.8%	8.4%	20.9%	19.7%	43.1%	3.8199	1.28807
I deworm the chickens or send off fecal samples for testing at the corresponding recommended time intervals	24.4%	7.8%	15.0%	21.6%	31.2%	3.2924	1.55905
Regardless of the cost, if there is a treatment that could help my chicken, then I pay for it or fundraise for it if I can’t afford it	20.1%	14.4%	18.5%	17.0%	30.1%	3.2145	1.50641
I include a probiotic supplement in my chickens water/feed	22.1%	9.1%	31.6%	22.0%	15.2%	2.9852	1.33748
** I prefer natural deaths at home for chickens over euthanasia	5.1%	7.7%	36.3%	23.2%	27.7%	2.3972	1.11645
I let my chickens inside my house if they wish to come in	42.9%	16.3%	19.3%	10.0%	11.6%	2.3162	1.39729
I let my chickens rest for long periods inside the house if they wish to	53.2%	15.6%	13.1%	7.5%	10.6%	2.0641	1.37609
I feed any eggs my chickens lay back to them	50.2%	17.3%	23.5%	6.2%	2.7%	1.9597	1.11095
I have a post-mortem carried out to determine the cause of death if the cause of death of one of my chickens is unknown	80.3%	9.5%	6.6%	1.6%	2.0%	1.3517	0.82761

** This item was reverse scored to reflect the general consensus amongst animal welfare scientists (and the definition of euthanasia) that euthanasia is preferable for an animal’s welfare as it shortens suffering prior to death.

**Table 2:** Respondents’ level of agreement with items of the CAS (Chicken Attitude Scale), including means and standard deviations. Note: ** indicates that these items have been reverse scored, as noted in Section 2.

	Strongly Disagree	Disagree	Undecided	Agree	Strongly Agree	Mean	Std. Deviation
It is morally wrong to participate in cockfighting in any way	2.3%	0.1%	0.3%	2.1%	95.1%	4.8749	0.63658
I sometimes get upset when I see chickens in cages	2.8%	2.0%	2.9%	22.2%	70.0%	4.5483	0.87142
** Basically, humans have the right to use chickens as we see fit	58.5%	24.8%	10.1%	4.4%	2.2%	4.3320	0.97434
** Chickens are morally less important than dogs	50.1%	26.2%	14.5%	6.1%	3.0%	4.1438	1.06792
** Breeding chickens for maximum egg production is morally acceptable	45.1%	23.7%	14.0%	7.5%	9.7%	3.8675	1.32224
It is morally wrong to breed chickens when millions of male chickens are unwanted and killed	6.7%	12.9%	29.6%	21.2%	29.6%	3.5493	1.22174
** I think it is perfectly acceptable for chickens to be raised for human consumption	18.2%	9.7%	22.1%	33.3%	16.7%	2.7990	1.33571
The slaughter of chickens should be immediately stopped even if it means some people will be put out of work	19.7%	32.2%	22.8%	8.3%	17.1%	2.7192	1.33828
** Sometimes it is necessary to clip chickens’ wings to prevent them jumping/flying too high	13.2%	11.7%	19.7%	37.4%	18.0%	2.6443	1.26818
** I t is morally acceptable to keep chickens for their eggs	3.8%	3.9%	10.9%	41.9%	39.5%	1.9059	0.99308

**Table 3:** Respondents’ level of agreement with OBRS (Owner–Bird Relationship Scale) items is shown. Note: ** indicates the item is reverse scored as noted in Section 2.

		Strongly Disagree	Disagree	Undecided	Agree	Strongly Agree
1	I enjoy playing with my chicken	2.9%	8.9%	13.8%	41.9%	32.4%
2	I think my chicken understands me	7.3%	19.8%	31.0%	30.9%	11.0%
3	My chicken knows when I am feeling bad	18.8%	32.1%	36.9%	8.1%	4.0%
4	I consider my chicken to be a friend	10.8%	21.1%	17.6%	33.4%	17.1%
5	My chicken is an equal member of my family	12.4%	25.7%	15.1%	29.4%	17.4%
6	Sometimes I wonder what my chicken is thinking	4.5%	8.3%	9.5%	48.0%	29.6%
7	I can talk to my chicken about anything	14.7%	17.2%	16.5%	28.4%	23.3%
8	My chicken is like a child to me	31.4%	29.3%	14.6%	15.2%	9.5%
9	My chicken provides structure for my life	13.8%	16.3%	17.0%	36.7%	16.2%
10	Having a chicken gives me someone to care for	10.2%	13.8%	18.1%	40.8%	17.2%
11	My chicken makes me feel needed	15.4%	21.7%	22.1%	28.4%	12.4%
12	Spending time with my chicken makes me forget my problems for a while	5.8%	7.1%	13.6%	44.2%	29.3%
13	I feel relaxed/more content because of my chicken	4.1%	5.9%	14.6%	44.7%	30.7%
14	I feel distressed when my chicken is ill and I see him/her suffering	1.5%	2.1%	4.0%	30.9%	61.6%
15	When my chicken is ill, it is my duty to care for him/her	0.3%	0.2%	0.7%	17.3%	81.5%
16	I pay attention to my chicken’s body language	0.5%	0.8%	5.8%	36.3%	56.6%
17	My chicken has his/her own unique personality	0.4%	0.7%	3.8%	25.1%	70.0%
18	My chicken is a sensitive being with his/her own needs	1.1%	2.0%	9.5%	30.4%	57.1%
19	My chicken actively tries to be close to me	4.8%	11.7%	23.4%	36.7%	23.5%
20	** My chicken always tries to keep a little distance from me	23.4%	33.3%	22.9%	18.5%	2.1%
21	** My chicken ignores me	50.0%	29.6%	14.2%	4.6%	1.6%

**Table 4:** PCA (with varimax rotation) item loading onto the four dimensions from Burmeister et al. [34], as indicated by the bold font, is shown. Note: Items loading 0.4 or higher are considered acceptable (in bold). The mean levels of agreement for respondents overall for each item of the OBRS (Owner–Bird Relationship Scale) and the standard deviations are also shown. Note: The item “Sometimes I wonder what my bird is thinking” loads onto two dimensions. Following advice from the original authors of the OBRS [44], this item was assigned to the first dimension in accordance with the original mapping of items onto dimensions in their study. The high similarity of the PCA results, the high Cronbach’s alphas (see below), and the negligible difference between the two dimension scores further contributed to this decision. ** indicates the items were reverse scored as noted in Section 2.

	Dimension					
	Bird as Human	Social Support	Empathy	Bird Reciprocity	Mean Level of Agreement	Std. Dev.
My chicken knows when I am feeling bad	**0.784**	0.156	0.053	0.107	**2.47**	**1.016**
I think my chicken understands me	**0.751**	0.115	0.126	0.128	**3.19**	**1.094**
I consider my chicken to be a friend	**0.738**	0.287	0.205	0.142	**3.27**	**1.259**
My chicken is an equal member of my family	**0.705**	0.273	0.257	0.082	**3.15**	**1.312**
My chicken is like a child to me	**0.624**	0.433	0.136	0.080	**2.44**	**1.323**
I can talk to my chicken about anything	**0.619**	0.373	0.221	0.044	**3.29**	**1.372**
I enjoy playing with my chicken	**0.538**	0.179	0.254	0.178	**3.93**	**1.025**
Having a chicken gives me someone to care for	0.244	**0.811**	0.132	0.007	3.42	1.211
My chicken provides structure for my life	0.299	**0.771**	0.122	0.043	3.26	1.286
My chicken makes me feel needed	0.339	**0.765**	0.110	0.032	3.02	1.267
Spending time with my chicken makes me forget my problems for a while	0.166	**0.720**	0.257	0.095	3.85	1.092
I feel relaxed/more content because of my chicken	0.186	**0.679**	0.303	0.122	3.93	1.013
When my chicken is ill, it is my duty to care for him/her	0.032	0.064	**0.778**	0.069	**4.80**	**0.466**
My chicken has his/her own unique personality	0.297	0.100	**0.711**	0.080	**4.64**	**0.626**
I pay attention to my chicken’s body language	0.184	0.175	**0.690**	0.137	**4.48**	**0.680**
I feel distressed when my chicken is ill and I see him/her suffering	0.056	0.218	**0.677**	0.051	**4.49**	**0.788**
My chicken is a sensitive being with his/her own needs	0.300	0.197	**0.661**	0.038	**4.41**	**0.816**
Sometimes I wonder what my chicken is thinking	**0.415**	0.259	0.470	0.002	**3.92**	**1.047**
** My chicken always tries to keep a little distance from me	0.128	−0.002	−0.022	**0.845**	3.57	1.090
** My chicken ignores me	0.054	0.028	0.127	**0.726**	4.22	0.950
My chicken actively tries to be close to me	0.328	0.241	0.213	**0.638**	3.63	1.102
Rotation Method: Varimax with Kaiser Normalization. Rotation converged in 6 iterations.						

**Table 5:** Shows a demonstration of significant differences in mean scores on the Care Series, CAS (Chicken Attitude Scale), and OBRS (Owner–Bird Relationship Scale) between different groups (types/style of carer). Note: D1–4 represent the different subscales (dimensions) of the OBRS. The test used is indicated in each section’s row heading. Bold face indicates significant differences (*p* < 0.05).

Scale	Care Series	CAS	OBRS				
Carer Type/Style			D1 (Bird as Human)	D2 (Social Support)	D3 (Empathy)	D4 (Bird Reciprocity)	Overall OBRS
Kruskal–Wallis Test							
Source most	0.333	**0.000**	0.143	0.655	**0.041**	**<0.001**	0.139
Hens, cocks, both	**<0.001**	0.765	0.504	0.579	0.362	**0.007**	0.794
Hens and cocks separate?	**0.024**	0.903	0.574	0.195	0.210	**0.030**	0.299
Primary caregiver?	0.197	0.189	0.592	0.490	0.592	0.636	0.775
Vaccinate?	**<0.001**	**0.021**	**<0.001**	**0.004**	**<0.001**	**<0.001**	**<0.001**
Name chickens?	**0.000**	**<0.001**	**0.000**	**0.000**	**0.000**	**<0.001**	**0.000**
Setup	**<0.001**	**0.004**	**<0.001**	0.110	**<0.001**	**0.005**	**<0.001**
Coop material	0.594	0.609	0.689	0.117	0.172	0.544	0.405
Follow lockdown?	**<0.001**	0.083	0.051	0.413	0.230	0.601	0.128
Kill to eat?	**<0.001**	**0.000**	**0.000**	**<0.001**	**<0.001**	**<0.001**	**0.000**
How tend to corpse?	**0.000**	**0.000**	**0.000**	**<0.001**	**0.000**	**0.001**	**0.000**
Killing method	0.698	0.869	0.209	0.324	0.174	0.543	0.417
Human diet	**0.000**	**0.000**	**0.000**	**<0.001**	**0.000**	**<0.001**	**0.000**
Human diet group	**0.000**	**0.000**	**0.000**	**<0.001**	**0.000**	**<0.001**	**0.000**
Memorialize?	**0.000**	**0.000**	**0.000**	**0.000**	**0.000**	**<0.001**	**0.000**
Take to vets?	**0.000**	**0.000**	**0.000**	**0.000**	**0.000**	**<0.001**	**0.000**
Home treatment success?	**<0.001**	0.211	0.108	0.121	0.201	**0.037**	**0.025**
Happy with vets?	**<0.001**	**0.002**	0.297	0.348	**0.048**	0.360	0.148
Role name	**0.000**	**0.000**	**0.000**	**<0.001**	**0.000**	**<0.001**	**0.000**
Consider implant?	**0.000**	**0.000**	**0.000**	**<0.001**	**<0.001**	**<0.001**	**0.000**
Implants improve lifespan	**<0.001**	**<0.001**	**<0.001**	**<0.001**	**<0.001**	0.065	**<0.001**
Implants improve life quality	**<0.001**	**<0.001**	**<0.001**	**0.006**	**<0.001**	0.630	**<0.001**
Regularly use implants	**<0.001**	**<0.001**	**<0.001**	**<0.001**	**<0.001**	**0.029**	**<0.001**
Approach if stop laying	**<0.001**	**<0.001**	**<0.001**	**0.006**	**<0.001**	**0.003**	**<0.001**
In will?	**0.000**	**<0.001**	**<0.001**	**<0.001**	**<0.001**	**<0.001**	**<0.001**
10 years’ time	**<0.001**	**<0.001**	**<0.001**	**<0.001**	**<0.001**	**<0.001**	**0.000**
Gender	**<0.001**	**<0.001**	**<0.001**	**<0.001**	**<0.001**	**0.033**	**<0.001**
Country	**0.001**	0.227	**<0.001**	**<0.001**	**<0.001**	**0.030**	**<0.001**
Home type	**0.009**	**0.013**	**<0.001**	**<0.001**	**0.016**	0.169	**<0.001**
Occupation	**<0.001**	**<0.001**	**<0.001**	**0.002**	**<0.001**	0.210	**<0.001**
Household profile	**<0.001**	**<0.001**	**<0.001**	**<0.001**	**<0.001**	**0.010**	**<0.001**
Mann–Whitney U Test							
Daily feces removal from run?	**0.000**	**<0.001**	**0.000**	**<0.001**	**<0.001**	**<0.001**	**0.000**
Heard of implant?	**0.000**	**0.000**	**0.000**	**<0.001**	**0.000**	**<0.001**	**0.000**
Cared for special needs?	**<0.001**	**<0.001**	**<0.001**	**<0.001**	**<0.001**	0.078	**<0.001**
Able to select closest chicken?	**0.000**	**<0.001**	**0.000**	**0.000**	**0.000**	**0.000**	**0.000**
Rent?	**<0.001**	0.440	**<0.001**	**<0.001**	0.076	**0.004**	**<0.001**
Kruskal–Wallis Test							
No. of chickens	**<0.001**	0.303	0.084	0.091	0.914	**0.003**	0.343
How long caring	**<0.001**	**<0.001**	**<0.001**	**0.010**	**<0.001**	**0.013**	**<0.001**
Stage of life	0.292	**0.000**	0.091	0.658	0.263	**<0.001**	0.070
Freq. coop feces removal	**0.000**	**0.000**	**0.000**	**<0.001**	**0.000**	**<0.001**	**0.000**
Run size	**0.007**	0.135	0.261	0.251	0.309	0.544	0.519
Human age	**<0.001**	**0.002**	**0.000**	**<0.001**	**<0.001**	**0.004**	**0.000**
Education level	**0.007**	0.190	**<0.001**	**<0.001**	0.616	0.782	**<0.001**
Living environment	0.881	0.277	0.161	**<0.001**	0.559	0.754	**0.022**

**Table 6:** Results of the Bonferroni post hoc test are shown, demonstrating which specific groups generated significant differences in scale scores for exemplar variables (*p* < 0.05). Note: Comprehensive results for each variable are available upon request.

	Type of Carer	Significant Care Series Score Differences	Significant CAS Score Differences	Significant OBRS Score Differences (D1–4 and Overall)
**Variable**	Consider implant use	Between all options bar “if more affordable” and “unsure”. “Yes” scored highest and “no” lowest.	Between “no” and all other options. “Yes” scored highest and “no” lowest.	D1–3 and Overall: Between all options bar “if more affordable” and “unsure”. D2 also not between “yes” and “if more affordable”. D4: Between “no” and “yes”, and “no” and “unsure”. All: “yes” scored highest and “no” lowest.
**Answers**	1. Yes, 2. Yes if more affordable, 3. Unsure, 4. No			
**Variable**	Diet group	Between all options. PB/vegan scored highest and omnivore lowest.	Between all options. PB/vegan scored highest and omnivore lowest.	Between all options. PB/vegan scored highest and omnivore lowest.
**Answers**	1. Omnivore, 2. Vegetarian, 3. PB/vegan *			
**Variable**	Source most	N/A (not significant)	Between: - Hatchery and all three rehoming options (4–6) - Local breeder and all three rehoming options (4–6) - Self-reared and all three rehoming options (4–6) “School hatching projects” highest score, followed closely by other two rehoming types (4, 6); hatchery scored lowest.	Only D3–4 significant. D3: Local breeder and ex-farm. D4: Self-reared and ex-farm. Local breeder and ex-farm. D3–4: Unwanted school hatching projects scored highest. D3: Hatchery scored lowest. D4: Self-reared scored lowest.
**Answers**	1. Hatchery 2. Local breeder 3. Self-reared 4. Ex-farm 5. Unwanted school hatching projects 6. Unwanted—friend/shelter			
**Variable**	Country	Between the UK and USA. “Other” scored highest, “NZ” lowest.	N/A (not significant)	D1: Between the UK and USA. “Other” scored highest, “NZ” lowest. D2: Between UK and USA. Canada scored highest, UK lowest. D3: Between UK and USA. Between UK and EU countries. Canada and Australia scored highest, NZ lowest. D4: Between Australia and Canada. Canada scored highest, Australia lowest.
**Answers**	1. USA, 2. Canada, 3. Australia, 4. NZ, 5. UK, 6. EU country, 7. Another European country, 8. Other			
**Variable**	Number of chickens	Between 3–6 and 13–20. 61+ scored highest, 1 lowest.	N/A (not significant)	Only D4 significant. Between 31–60 and 2. 2 scored highest, 31–60 lowest.
**Answers**	1, 2, 3–6, 7–12, 13–20, 21–30, 31–60, 61+			
**Variable**	Name chickens	Between all options. “Yes” scored highest, “no” lowest.	Between “no” and “yes”, and “some” and “yes”. “Yes” scored highest, “no” lowest.	D1: Between all options. D2: Not between “no” and “some”. D3: Between all options. D4: Not between “no” and “some”. Overall: Between all options. All: “Yes” scored highest, “no” lowest.
**Answers**	No, Yes, Some			
**Variable**	Vet use	Between all options. “Exotic” scored highest, “no” lowest.	Between all options. “Exotic” scored highest, “no” lowest.	All (bar D4): Between all options. D4: Between all options bar: “treat at home” and “standard”, and “treat at home” and “no”. All: “Exotic” scored highest, “no” lowest.
**Answers**	1. Exotic, 2. Standard, 3. Treat at home, 4. No			

* To simplify the table, the diet “other” answer option was removed.

**Table 7:** Spearman’s rho correlation coefficients regarding correlation between the OBRS (Owner-Bird-Relationship Scale)/CAS (Chicken Attitude Scale) and individual Care Series items are shown. Note: D1–4 represents the different subscales (dimensions) of the OBRS. Correlation coefficients over 0.4 are highlighted in yellow; those over 0.2 are shown in green. * signifies *p* < 0.05, ** signifies *p* < 0.001.

Scale	CAS	D1 Bird as Human	D2 Social Support	D3 Empathy	D4 Bird Reciprocity	OBRS Overall
Care Item						
I worry about my chickens’ health and wellbeing	0.302 **	0.341 **	0.318 **	0.386 **	0.121 **	0.374 **
I find caring for chickens easy	−0.118 **	−0.059 **	−0.051 *	−0.067 **	0.042	−0.048 *
I find caring for chickens stressful	0.175 **	0.090 **	0.098 **	0.147 **	−0.034	0.097 **
I prefer natural deaths at home for chickens over euthanasia	−0.075 **	−0.046 *	−0.061 **	−0.106 **	−0.026	−0.074 **
I conduct periodical health checks of my chickens	0.087 **	0.245 **	0.181 **	0.244 **	0.193 **	0.270 **
I ensure my chickens have fresh material for dustbathing in	0.097 **	0.137 **	0.112 **	0.187 **	0.143 **	0.167 **
I have a post-mortem carried out to determine the cause of death if the cause of death of one of my chickens is unknown	0.079 **	0.154 **	0.099 **	0.125 **	0.083 **	0.148 **
I deworm the chickens or send off fecal samples for testing at the corresponding recommended time intervals	0.110 **	0.159 **	0.155 **	0.186 **	0.126 **	0.198 **
I let my chickens inside my house if they wish to come in	0.274 **	0.479 **	0.302 **	0.357 **	0.207 **	0.458 **
I let my chickens rest for long periods inside the house if they wish to	0.235 **	0.442 **	0.291 **	0.334 **	0.190 **	0.426 **
Take my chicken on trips	0.118 **	0.202 **	0.122 **	0.123 **	0.102 **	0.186 **
I spend time observing my chickens’ behavior	0.181 **	0.361 **	0.350 **	0.417 **	0.195 **	0.419 **
Use of chicken diaper	0.124 **	0.175 **	0.145 **	0.117 **	0.053 *	0.168 **
I arrange for someone to care for my chickens when I am away for a weekend	0.044 *	0.022	−0.014	0.068 **	0.045 *	0.029
Use of chicken sweater	0.038	0.120 **	0.033	0.042	0.002	0.089 **
I try to improve my knowledge of optimal care-taking practices for chickens	0.178 **	0.281 **	0.264 **	0.364 **	0.161 **	0.332 **
Give eggs back to hens	0.254 **	0.247 **	0.208 **	0.230 **	0.074 **	0.254 **
Use of chicken leash	0.064 **	0.075 **	0.068 **	0.035	0.012	0.067 **
I introduce new chickens to a flock gradually over a period of a few weeks	0.108 **	0.082 **	0.087 **	0.142 **	0.049 *	0.111 **
I clean my chickens’ hind feathers if they become very soiled	0.184 **	0.267 **	0.184 **	0.273 **	0.128 **	0.277 **
I include a probiotic supplement in my chickens water/feed	0.133 **	0.197 **	0.122 **	0.176 **	0.112 **	0.195 **
I include a calcium supplement in my chickens water/feed	0.076 **	0.114 **	0.077 **	0.111 **	0.074 **	0.116 **
Regardless of cost, pay/fundraise for vet treatment	0.352 **	0.451 **	0.312 **	0.420 **	0.196 **	0.457 **
Caring harder than thought	0.087 **	0.067 **	0.053 *	0.050 *	−0.057 **	0.051 *
Confident providing excellent care	0.002	0.157 **	0.109 **	0.165 **	0.121 **	0.172 **

**Table 8:** Spearman’s rho correlation coefficients for the mean scores on the OBRS (Owner–Bird Relationship Scale) with mean CAS (Chicken Attitude Scale) scores and Care Series scores are shown. Note: Correlation coefficients 0.4 and above are emboldened. *p* < 0.001.

	Mean Score: Bird as Human	Mean Score: Social Support	Mean Score: Empathy	Mean Score: Bird Reciprocity	Mean Score: Overall OBRS	Mean Care Series Score
Mean CAS score	0.363	0.231	**0.400**	0.176	0.374	0.351
Mean Care Series score	**0.515**	0.382	**0.500**	0.263	**0.543**	**-**

## Data Availability

The data acquired in this project can be made available upon request.

## Supplementary Materials

The following are available online at https://www.mdpi.com/article/10.3390/ani14020288/s1, Supplementary Materials A: Consent form and questionnaire; Supplementary Materials B: Figure S1–S6; Supplementary Materials C: Table S1–S13; Supplementary Materials D: Discussion of results that supplement extant literature. Refs. [76,77,78,79,80,81,82,83,84] are cited in the Supplementary Materials.

## References

[1] ChickenGuard (2020). How to Keep Chickens—Can You Keep Chickens in the UK?. https://www.chickenguard.co.uk/how-to-keep-chickens-can-you-keep-chickens-in-the-uk/.

[2] Everett F. (2020). Move over Dogs and Cats, Chickens Are Becoming Our New Favourite Pets. The Telegraph.

[3] Spots.com (2023). Pet Ownership Statistics. https://spots.com/pet-ownership-statistics/.

[4] AVMA U.S (2023). Pet Ownership Statistics. https://www.avma.org/resources-tools/reports-statistics/us-pet-ownership-statistics.

[5] Statista (2023). Number of Households in the U.S. from 1960 to 2022. https://www.statista.com/statistics/183635/number-of-households-in-the-us/.

[6] APPA [American Pet Product Association] (2022). The 2021–2022 APPA National Pet Owners Survey.

[7] Megna M. Pet Ownership Statistics 2023. *Forbes Advisor*; 2023. https://www.forbes.com/advisor/pet-insurance/pet-ownership-statistics/.

[8] MRCVSonline (2020). Fears over Surge in Abandoned “Lockdown” Chickens. https://mrcvs.co.uk/en//news/20011/Fears-over-surge-in-abandoned-%27lockdown%27-chickens.

[9] Verdolini G. Meghan Markle and Prince Harry Rescue Chickens and Show Off Their Coop. *The Beet*; 2021. https://thebeet.com/meghan-markle-prince-harry-rescue-chickens-and-show-off-their-coop/.

[10] BHWT (2023). We’re the British Hen Welfare Trust, a Charity Committed to Changing the Lives of Commercial Laying Hens since 2005. https://www.bhwt.org.uk/.

[11] Dogs Trust (2023). Our impact. https://www.dogstrust.org.uk/about-us/accounts-annual-reviews.

[12] Champs Libres Aux Poules (2021). Champs Libres Aux Poules. https://www.champslibresauxpoules.com/.

[13] Ayala A.J., Yabsley M.J., Hernandez S.M. (2020). A review of pathogen transmission at the backyard chicken–wild bird interface. Front. Vet. Sci..

[14] Correia-Gomes C., Sparks N. (2020). Exploring the attitudes of backyard poultry keepers to health and biosecurity. Prev. Vet. Med..

[15] Kyle C., Sutherland L.-A. (2018). Understanding Backyard Poultry Keepers and Their Attitudes to Biosecurity: Preliminary Report.

[16] Lockhart C.Y., Stevenson M.A., Rawdon T.G. (2010). A cross-sectional study of ownership of backyard poultry in two areas of Palmerston North, New Zealand. N. Z. Vet. J..

[17] Zoubek E. (2018). From Egg to Dead: Small-Scale Chicken Keeping in Modern Britain. Ph.D. Thesis.

[18] Souvestre M., Delpont M., Guinat C., Dumat C., Guichard L., Manis L., Duret H., Guérin J.L., Le Loc’h G. (2021). Backyard poultry flocks in France: A diversity of owners and biosecurity practices. Prev. Vet. Med..

[19] Singleton D.A., Ball C., Rennie C., Coxon C., Ganapathy K., Jones P.H., Welchman D., Tulloch J.S. (2021). Backyard poultry cases in UK small animal practices: Demographics, health conditions and pharmaceutical prescriptions. Vet. Rec..

[20] Demetriou S.C. (2021). Cultivating Care: Backyard Hens and the Changing Geography of Human-Chicken Relations in Toronto. Master’s Thesis.

[21] Karabozhilova I., Wieland B., Alonso S., Salonen L., Häsler B. (2012). Backyard chicken keeping in the Greater London Urban Area: Welfare status, biosecurity and disease control issues. Br. Poult. Sci..

[22] Danovich T. (2023). Under the Henfluence.

[23] Open Sanctuary (2020). Suprelorin Implants. https://opensanctuary.org/article/suprelorin-implants-a-critical-tool-in-chicken-health/.

[24] Elkhoraibi C., Blatchford R.A., Pitesky M.E., Mench J.A. (2014). Backyard chickens in the United States: A survey of flock owners. Poult. Sci..

[25] Pohjola L., Rossow L., Huovilainen A., Soveri T., Hänninen M.L., Fredriksson-Ahomaa M. (2015). Questionnaire study and postmortem findings in backyard chicken flocks in Finland. Acta Vet. Scand..

[26] Singleton D.A., Sánchez-Vizcaíno F., Arsevska E., Dawson S., Jones P.H., Noble P.J.M., Pinchbeck G.L., Williams N.J., Radford A.D. (2018). New approaches to pharmacosurveillance for monitoring prescription frequency, diversity, and co-prescription in a large sentinel network of companion animal veterinary practices in the United Kingdom, 2014–2016. Prev. Vet. Med..

[27] Taggers A., Baron H. (2023). A retrospective study of 333 emergency presentations and survival to discharge of backyard chickens in Australia from 2019 to 2022. Aust. Vet. J..

[28] Vaught M.E., Gladden J.N., Rozanski A.N. (2019). Reasons for evaluation on an emergency basis of and short-term outcomes for chickens from backyard flocks: 78 cases (2014–2017). J. Am. Vet. Med. Assoc..

[29] Arluke A., Sanders S. (1996). Regarding Animals.

[30] Macauley L. (2018). Friends, Food, or “Free Egg Machines”? A Qualitative Study of Chicken Owners’ Perceptions of Chickens and Chicken Meat. BSc Thesis.

[31] Oliver C. (2021). Returning to “The Good Life”? Chickens and chicken-keeping during COVID-19 in Britain. Anim. Stud. J..

[32] Herzog H., Grayson S., McCord D. (2015). Brief measures of the animal attitude scale. Anthrozoos.

[33] Robertson J.C., Gallivan J., Maclntyre P.D. (2004). Sex differences in the antecedents of animal use attitudes. Anthrozoos.

[34] Burmeister A.K., Drasch K., Rinder M., Prechsl S., Peschel A., Korbel R., Saam N.J. (2020). Development and application of the Owner-Bird Relationship Scale (OBRS) to assess the relation of humans to their pet birds. Front. Vet. Sci..

[35] Macauley L., Chur-Hansen A. (2023). Human health benefits of non-conventional companion animals: A narrative review. Animals.

[36] Blatchford R.A., Mench J.A. (2017). Backyard flock production. Advances in Poultry Welfare.

[37] Lister S.A. (2021). Opportunities and challenges in backyard poultry health and management. Vet. Rec..

[38] Eusemann B.K., Sharifi A.R., Patt A., Reinhard A.K., Schrader L., Thöne-Reineke C., Petow S. (2018). Influence of a sustained release deslorelin acetate implant on reproductive physiology and associated traits in laying hens. Front. Physiol..

[39] Abou-Zahr T. (2022). Avian reproductive disorders. Companion Anim..

[40] Davies G. (2016). Common ailments of pet hens. Vet. Nurs. J..

[41] Greenacre C.B. (2015). Reproductive diseases of the backyard hen. J. Exot. Pet Med..

[42] BHWT (2023). What Is Egg Yolk Peritonitis?. https://www.bhwt.org.uk/blog/health-welfare/egg-yolk-peritonitis/.

[43] Online Surveys (2019). About Online Surveys. https://www.onlinesurveys.ac.uk/about/.

[44] Saam N., Rinder M., Drasch K. (2022). Personal communication.

[45] SurveyMonkey Sample Size Calculator. n.d. https://www.surveymonkey.co.uk/mp/sample-size-calculator/.

[46] Architecture & Design (2021). How Many Houses Are in the World?. https://www.architectureanddesign.com.au/features/list/how-many-houses-are-in-the-world.

[47] Bryman A. (2016). Quantitative data analysis. Social Research Methods.

[48] Field A. (2013). Discovering Statistics Using IBM SPSS Statistics.

[49] Marchant-Shapiro T. (2015). Chi-square and Cramer’s V: What do you expect?. Statistics for Political Analysis: Understanding the Numbers.

[50] McHugh M.L. (2013). The chi-square test of independence. Biochem. Med..

[51] Jayaratne S., Tripodi T., Talsma E. (1988). The comparative analysis and aggregation of single-case data. J. Appl. Behav. Sci..

[52] Weymouth A. (2022). Using hardwood woodchip for your chicken run. Flyte So Fancy.

[53] Roberts V. (2020). How to navigate through the regulations and medications for backyard and pet poultry. Livestock.

[54] Joy M. (2010). Why We Love Dogs, Eat Pigs, and Wear Cows: An Introduction to Carnism.

[55] Mace J.L., McCulloch S.P. (2020). Yoga, ahimsa and consuming animals: UK yoga teachers’ beliefs about farmed animals and attitudes to plant-based diets. Animals.

[56] Gopnik A., Griffiths T.L., Lucas C.G. (2015). When younger learners can be better (or at least more open-minded) than older ones. Curr. Dir. Psychol. Sci..

[57] FDA (2023). Suprelorin® F (Deslorelin Acetate). https://www.fda.gov/media/83933/download.

[58] Rosenzweig A. (2022). Ask FDA to Approve Chicken Implants to Prevent Egg Laying (Deslorelin). https://www.change.org/p/ask-fda-to-approve-chicken-implants-to-prevent-egg-laying-deslorelin?utm_content=cl_sharecopy_34293796_en-US%3A9&recruited_by_id=163bf820-2495-11ee-b860-5dd9390be998&utm_source=share_petition&utm_medium=copylink&utm_campaign=psf_combo_share_initial&share_bandit_exp=skip-34293796-en-GB.

[59] YouGov (2023). Dietary Choices of Brits. https://yougov.co.uk/topics/society/trackers/dietery-choices-of-brits-eg-vegeterian-flexitarian-meat-eater-etc.

[60] Statista Research Department (2023). Veganism and Vegetarianism in the United States—Statistics & Facts. https://www.statista.com/topics/3377/vegan-market/#topicOverview.

[61] Dodd S.A., Cave N.J., Adolphe J.L., Shoveller A.K., Verbrugghe A. (2019). Plant-based (vegan) diets for pets: A survey of pet owner attitudes and feeding practices. PLoS ONE.

[62] Donaldson S., Kymlicka W. (2011). Zoopolis: A Political Theory of Animal Rights.

[63] Wolf A. (2021). The Ethics of Proximity: A Defense of Different Ethical Duties to Friends and Family. Epoché.

[64] Pollock C. (2016). Backyard poultry in clinical avian practice. J. Avian Med. Surg..

[65] Hess T., Magnus J. (2021). Sharing Your Home with Chickens. The Open Sanctuary Project. https://opensanctuary.org/indoorchickens/.

[66] Mellor D.J. (2016). Updating animal welfare thinking: Moving beyond the “five freedoms” towards “A life worth living”. Animals.

[67] Yilmaz Dikmen B. Laying hen behaviour and welfare in housing systems. Proceedings of the 25th International Scientific Experts Congress on Agriculture and Food Industry.

[68] Henke J. (2021). Stop Chickens from Eating Eggs. https://www.agriculture.com/podcast/living-the-country-life-radio/stop-chickens-from-eating-eggs.

[69] Calder C., Albright J., Morishita T.Y., Greenacre C.B. (2021). Chicken behaviour. Backyard Poultry Medicine and Surgery.

[70] Open Sanctuary (2018). What Should a Sanctuary do with Residents’ Eggs?. https://opensanctuary.org/what-to-do-about-egg-laying/.

[71] Steele L. (2023). Myths about feeding your chickens eggshells. Fresh Eggs Daily.

[72] The Chicken Chick® (2023). Chicken Sweaters: Just Say No. https://the-chicken-chick.com/chicken-sweaters-just-say-no/.

[73] Burmeister A.K., Drasch K., Rinder M., Prechsl S., Peschel A., Korbel R., Saam N.J. (2022). The owner-bird relationship: Relevance for pet bird welfare. Anim. Welf..

[74] Magnus J., Griffler M. (2023). So You Want to Rescue a Rooster (Brochure). The Open Sanctuary Project. https://opensanctuary.org/the-open-sanctuary-projects-so-you-want-to-rescue-a-rooster-brochure/.

[75] BHWT (2023). Cockerel Adoption. https://www.bhwt.org.uk/cockerel-adoption/.

[76] UK Government (2007). The Mutilations (Permitted Procedures) (England) Regulations 2007. https://www.legislation.gov.uk/uksi/2007/1100/schedule/4/made.

[77] Kirk I. (2022). Should Selective Breeding of Dogs with Health Issues Be Banned? YouGov. https://yougov.co.uk/topics/society/articles-reports/2022/04/13/should-selective-breeding-dogs-health-issues-be-ba.

[78] Kodilinye-Sims H., Royden A. (2022). We must improve our pet poultry services. Vet. Rec..

[79] ECDC [European Centre for Disease Prevention and Control] (2022). 2021–2022 Data Show Largest Avian Flu Epidemic in Europe Ever. https://www.ecdc.europa.eu/en/news-events/2021-2022-data-show-largest-avian-flu-epidemic-europe-ever.

[80] Nicol C.J. (2015). The Behavioural Biology of Chickens.

[81] BHWT (2023). Merging Your Flocks. https://www.bhwt.org.uk/merging-your-flocks/.

[82] HSA (2023). Electrical Stunning Equipment. https://www.hsa.org.uk/stunning-and-slaughter-electrical-stunning/equipment-3.

[83] HSA (2023). Neck Dislocation. https://www.hsa.org.uk/neck-dislocation/neck-dislocation.

[84] HSA (2023). Other Methods. https://www.hsa.org.uk/other-methods/other-methods.

