# Supplementary material for: From the Backyard to Our Beds: The Spectrum of Care, Attitudes, Relationship Types, and Welfare in Non-Commercial Chicken Care
Source: Animals (Basel). 2024 Jan 17;14(2):288. doi: 10.3390/ani14020288 (PMC10812673; doi:10.3390/ani14020288)
Supplement: Supplementary file 1 [file animals-14-00288-s001.zip › animals-2804038-supplementary.pdf]

Supplementary Materials – Contents

A – Consent form and questionnaire 2
B – Figures 22
C – Tables 25
D – Extra discussion 40

Supplementary Materials A. Consent form and questionnaire

Information and consent form

Introduction

This questionnaire is for those who look after chickens in a non-commercial capacity, meaning no funds are generated from the chickens. You must be 18 or older and have the chickens permanently in your care (i.e., not just fostering for short periods).

The questionnaire will take around 20 minutes to complete. The questionnaire forms part of a study that aims to collect much-needed data on the care-taking practices and attitudes towards chickens of non-commercial chicken carers. The research is being conducted by Jenny L. Mace, an ex-commercial chicken carer and remote visiting lecturer with The Centre for Animal Welfare at the University of Winchester, UK.

The questionnaire is split into six sections, each of which focuses on the following:

1) Demographic information about your chickens
2) Your chicken care-taking practices and experiences
3) The relationship you have with your chickens
4) Your attitudes towards chickens
5) The future of your chicken care
6) Demographic information about you

Please ONLY answer this survey if you look after chickens in a non-commercial capacity (i.e., you do not make any funds from your chickens).

Participation and Prize Draw

Participation in this survey is voluntary. Participants are free to opt out at any time during the survey and can do so by navigating away from the page. If you do not provide an email address when invited to do so, you will be unable to withdraw your data after submitting it as it will be unidentifiable. If you do provide your email address, you will be free to withdraw your data up to 24 hours after submission because it is at this point that your data will become anonymized. By supplying an email address, you will also be entered into the prize draw for a £50 voucher redeemable at the British Hen Welfare Trust. The intention is to publish the results of this research in a peer-reviewed academic journal. Participating in this research will help to advance knowledge on the care-taking practices of non-commercial chicken carers and their relationships with their chickens. This is an underexplored area and caring for chickens, including ex-commercial chickens, is a growing phenomenon.

Data Protection

The data gathered will be anonymous and require no identifying factors such as name or email address, unless the participant volunteers this in the final question. In such instances, the data will be anonymized 24 hours after closing the survey or before the researcher examines any of this particular data. Data collected will be used solely for the purposes of this study and publications resulting from it. Data will be treated in accordance with The Data Protection Act 2018; that is, any names/email addresses will only be used for the purpose of disseminating links to the research and they will be deleted after this has been completed. Names and email addresses will also be stored separately from questionnaire

responses using a University-provided, secure, password-protected site.

Ethics

Ethical approval for this study has been granted by the University of Winchester.

Contact

Please use the following contact details if you require any further information about the project:

Jenny L. Mace, part-time remote lecturer – jenny.mace@winchester.ac.uk

If you have any concerns or complaints about this research, please contact the University Chair of Ethics at ethics@winchester.ac.uk.

Please select the statement below to confirm you have read the information above about the study and you consent to your data being used anonymously in the ways described.

○ Yes, I understand, agree with, and consent to the information above

Section 1: Chicken demographics

1. How many separate flocks of chickens are normally in your care? Please include any individual hens who don't live with other chickens as their own flock.

1
2
3
More than 3

2. How many chickens (counting all individuals in every flock if applicable) are typically in your care?

1 2 3-6 7-12 13-20 21-30 31-60 61 or more

3. Please select the option that best applies to you regarding how you source your chickens:

a. Most of the chickens in my care come from a hatchery
b. Most of the chickens in my care come from a local breeder
c. I breed and rear most of the chickens in my care myself
d. Most of the chickens in my care are ex-commercial chickens spared from slaughter
e. Most of the chickens in my care are unwanted chickens of neighbours or friends or in animal shelters
f. Other (please state)

<u>Question just for those opting for 3d and 3e</u>: If chickens did not need re-homing/rescuing, do you think you would still keep chickens?

Yes No Unsure

<u>Questions just for those opting for 19d</u>:

Do you know the farm type from which most of your chickens came?

Organic
Free-range
Barn
Cage (enriched or unenriched)
Not sure
Other (please state)

Do you know what the primary use of your chickens was in the former commercial setting?

For their eggs
For their meat
Both for their meat and eggs
Unsure

How do you acquire your ex-commercial chickens? You can select multiple answers if applicable.

- *Through a charity that rehomes former laying hens to prevent them going to slaughter*
- *Through participation in the Animal Save Movement and truck drivers outside slaughterhouses letting activists keep a few chickens*
- *Through being at the scene of a road traffic accident involving a truck of chickens and rescuing some*
- *Other*

<u>Question for those opting for 3a-c and 3e-f</u>: Would you ever consider re-homing ex-commercial chickens?

Yes No

Please explain your answer.

<u>Question for those opting for 3a-d and 3f</u>: Would you ever consider re-homing unwanted chickens (e.g. from a neighbour or in an animal shelter)?

Yes No

Please explain your answer.

4. Which of these options best applies to you?

a. I primarily care for hens
b. I primarily care for roosters/cocks
c. I care for both hens and roosters/cocks

<u>Question for those selecting 4a</u>: What is the reason you primarily care for hens and not roosters/cocks? You can select multiple answers if applicable.

They are too noisy for me
They are too noisy for my neighbours
It is not technically allowed to keep roosters/cocks in my area
I don't know enough about caring for roosters/cocks
I don't have space for a separate flock of roosters/cocks
I don't like roosters/cocks as much as hens
I find roosters/cocks too aggressive
I keep chickens for their eggs, so do not want a rooster/cock as I am not breeding chickens
Other

<u>Question for those selecting 4b</u>: What is the reason you primarily care for roosters/cocks and not hens? You can select multiple answers if applicable.

I prefer roosters/cocks to hens
I am horrified by the way roosters/cocks are treated commercially
The secluded environment I live in affords me the ability to care for them without disturbing neighbours
Roosters/cocks are more unwanted, neglected and abused in society than hens
Other

<u>Question for those selecting 4c</u>: Do you keep your hens and roosters/cocks together or in separate flocks?

Together
In separate flocks
Some together and some in separate flocks

If applicable, respondents are also asked: How many roosters/cocks do you keep with your hens? (1, 2, 3, 4, or more).

5. What breed or type are the majority of your chickens?
Hybrid/ISA Brown
Cornish cross
Leghorn
Light Sussex
Black Rock
Maran
Rhode Island Red
Bantam
Brahma
Orpington
Plymouth Rock
Ameraucana / Araucana / Easter Egger

Unsure
Other (please state)

Section 2: Chicken care-taking practices and experiences

6. What was the primary motivation for you to care for chickens? You can select multiple answers if applicable.

To source eggs in a higher welfare/more ethical capacity
To source chicken meat in a higher welfare/more ethical capacity
To educate children about food
To educate children about animals
To entertain children
To prevent commercial chickens going to slaughter
Self-sufficient food production at home
As a form of therapy
Covid-inspired
Other

7. Are you the primary caregiver for your chickens?

Yes
No
50:50 with another adult
My children do all the care-giving and I oversee it
Other

8. Do you vaccinate your chickens?

Yes No The farm/previous owners do so

9. Do you name your chickens?

Yes No Some

10. What is the typical age of your chickens when they come into your care? If the age varies, please select the most typical age.

I hatch them
As chicks (less than six months of age)
At sexual maturity
At around 18 months old or similar when farms would otherwise send their laying hens to slaughter
Two or older

11. Which of the following options best describes the kind of setup you have regarding how you keep your chickens? NB: For the purposes of this questionnaire, 'coop' refers to an indoor henhouse where chickens often sleep and 'run' refers to an outside enclosed area in which chickens can move around.

No coop or run: completely free-range
Just coop for overnight and completely free range during the day

Coop and run with free-range access a lot of the time
Coop and run with only supervised free-range access
Coop and run: no free-range allowed
My chicken(s) lives predominantly indoors in my home
Other

Questions for those selecting a coop:

What bedding do you normally use in the coop (i.e., the enclosed area where the chickens sleep)?

Straw / chopped straw
Hay
Aubiose (hemp shavings)
Wood shavings
Soft woodchip
Hard woodchip
Shredded paper
No bedding
Other (please state)

What is the material of your coop (i.e., the enclosed area where the chickens sleep)?

Wood *Plastic* *Metal* *Other*

How often do you remove faeces from your coop (i.e. the enclosed area where the chickens sleep)?

Daily
Every few days
Weekly
Never
Never – I use the deep litter method
Other

Questions for those selecting a run:

What substrate do you normally use in the run (i.e., the enclosed area in which chickens can move around in the open air)?

None - bare earth
Soil/compost
Grass
Straw
Hay
Hard woodchip
Soft woodchip
Rubber chipping
Other

Do you remove faeces that you can see from the run daily (i.e., the enclosed area in which chickens can move around in the open air)?

Yes No

What is the approximate size of your run (i.e., the enclosed area in which chickens can move around in the open air)?

Less than 2m x 2m
Between 2m x 2m and under 4m x 4m
Between 4m x 4m and under 6m x 6m
Between 6m x 6m and under 8m x 8m
More than 8m x 8m
Other

Question for those selecting coop and/or run:

Do you use an automatic door?

Yes No

Question for those selecting 'no free-ranging time at all': What is the reason you do not allow free-range access at all? You can select multiple answers if applicable.

To prevent the chickens from damaging the rest of my garden
I am too scared of predators and insufficient time/inclination to supervise the chickens if they were to free-range
I don't wish to get the chickens used to having free-range access, otherwise they may want it all the time
Other

12. Did you follow the legal requirements regarding keeping chickens inside/out of access to wild birds during the last avian flu 'chicken lockdown' in your area?

Yes
No
Partially
There has not been an avian flu 'chicken lockdown' in my area
Other

For those selecting Yes/Partially: Were you concerned the about your chickens' welfare during the lockdown?

Yes No

13. What measures have you taken to minimise predation risk to your chickens? You can select multiple answers if applicable.

- Only allowing supervised free-ranging time
- Placing the coop and run very close to the backdoors of our house
- Spending most of the day in a room where I can directly see my chickens

- Choosing galvanised hardware cloth/wire mesh rather than chicken wire for the run material
- Using electric fencing
- Having overhead netting/roof to their run
- Extending the sides of the run/mesh down into the earth by at least 30cm around the entire perimeter of the run
- Using 'skirting' around the entire perimeter of the run that extends out along the ground and is fixed to the ground
- Keeping chickens in a secure coop overnight
- Keeping chickens in a secure coop within a secure run overnight
- Covering the coop air vents with wire gauze
- Conducting regular checks of the integrity of the run/coop security
- Installing a webcam in the coop/enclosure
- The chickens sleep in my house at night
- None
- Other

14. What do you consider to be the lowest temperature that chickens can cope well with?

Below -10°C / 14°F

-10°C / 14°F

-5°C / 23°F

0°C / 32°F

5°C / 41°F

15. Please select all health conditions that any of your chickens has experienced within the last year.

No health problems in the last 12 months

Red mite

Feather lice

Worms

Marek's disease

Coccidiosis

Newcastle disease

Infectious bronchitis

Diarrhoea

Egg-binding

Egg yolk peritonitis

Respiratory problems (e.g. nasal or eye discharge, coughing, sneezing)

Neurological problems (e.g. weakness or co-ordination difficulties)

Weight loss

Lameness

Injuries

Foot pad dermatitis / bumblefoot

Scaly leg
Unknown health conditions
Other

16. Are you aware of any abnormal behaviour exhibited by any of your chickens (e.g. very persistently aggressive behaviour, persistent feather pecking or pacing)?

Yes (please describe)   No

17. Which of the following options most accurately reflects the typical weight of your chickens?

Most of my chickens weigh around 1.5kg
Most of my chickens weigh around 2kg
Most of my chickens weigh around 2.5kg
Most of my chickens weigh around 3kg
I do not know the weight of my chickens
Other

18. Do you find that your chickens' hind feathers are regularly soiled to such an extent that it is hard to manage?

Yes   Sometimes   No

19. How many of your chickens have died in the last year?

0   1   2   3   4   5   6   7+

20. On average, what age have your chickens lived to thus far?

Less than 3 years of age
3-5 years of age
6-8 years of age
9-11 years of age
A mix of these
Too early to tell
Other

21. What was the age of your chicken who has lived the longest thus far?

<3
3
4
5
6
7
8
9
10
11+

22. What have been the causes of death in your chickens (either with or without the aid of euthanasia)? You can select multiple answers if applicable.

Egg yolk peritonitis
Egg binding
Ovarian cancer/tumour
Another form of cancer/tumour
Crop disorder
Marek's disease
Newcastle disease
Coccidiosis
Respiratory disease
Predation by other animals
Predation by humans (me/family member) for food
Re-occurring prolapse
Heart failure
Theft (with belief that they will be killed or used in a blood sport)
Aggression from another chicken
Sudden death
Unknown cause
Other

23. Do you, or would you ever, kill your chickens for human consumption?

Yes   No   Yes, but I would ask a friend or other family member to do the killing for me

Question for those selecting 'yes': What method do/would you use to kill your chickens?

Neck wringing (i.e. cervical dislocation)
Killing cone/other method involving the cutting of neck veins or arteries
Decapitation
A mix of the above
Other

Question for those selecting 'no': How do you usually tend to your chickens' bodies when they die? If you have not experienced the death of any of your chickens yet, please imagine what you might do.

Bury on premises
Place in rubbish bin
Outsource cremation services
Cremate/burn at home
Other

24. Do you memorialise your chickens when they die (e.g., by having symbols or places of remembrance)?

Yes   No   Sometimes

25. Do you take your chickens to a vet when they are ill?

a. Yes, a standard non-exotics vet
b. Yes, an exotics vet
c. I mostly treat ill chickens at home using herbal/alternative remedies
d. I don't seek any treatment for ill chickens

<u>Extra questions for those selecting 25a</u>:

Which of the following options best describes your reasoning for not choosing an exotic vet? You can select multiple answers if applicable.

I do not know of one
My nearest exotic vet is too far away
Exotic vets are too expensive
There is an experienced avian vet at my standard vets / my vet practice is considered 'chicken-friendly'
Not sure what an exotic vet is
Other

<u>Extra question for those selecting 25a/b</u>:

Are you happy with the service your chickens receive at the vets?
Yes
No
Unsure

If not, why not?

<u>Extra questions for 25b</u>:

Which option best describes how long it takes you to reach your exotic vet (one way)?

Less than 15 minutes
Between 15 and under 30 minutes
Between 30 minutes and under 60 minutes
Between 1 and under 1.5 hours
Between 1.5 hours and under 2 hours
2 or more hours

<u>Extra questions for 25c</u>:

Have you had success in treating some health problems at home? (Yes/No/Partially)

What is your reasoning for treating chickens at home? You can select multiple answers if applicable.

Vets are too expensive
I believe in herbal remedies / other alternative health treatments
I fear vets will not know how to treat my chicken and will just suggest euthanasia
Other

26. Which of the following terms do you use to describe your chicken caring role?
Chicken rescuer
Chicken carer
Chicken keeper
Backyard farmer
Other (please state)

27. What chicken care resource do you most frequently use?
Webinars by specialists (please state an example)
Talking with other chicken carers
My vet
Forums or online groups (please state an example)
Other (please state)
None

28. Have you ever cared for a chicken who primarily lived indoors in your personal home?
a. Yes / b. No

Extra questions for those selecting 28a:

Please describe what motivated this step.

Extra questions for those selecting 28b:

Would you ever consider caring for a chicken who lives primarily in your home? (Yes/No)

If not, please explain why.

29. Have you heard of the Suprelorin hormonal implant that can be used in hens?
a. Yes / b. No

Extra questions for those selecting 29a:

Do you think the Suprelorin hormonal implant can improve the LENGTH of a hen's life? (Yes/No/Unsure)

Do you think the Suprelorin hormonal implant can improve the QUALITY of a hen's life? (Yes/No/Unsure)

Do you use the Suprelorin hormonal implant regularly with your hens?

- *Yes, for all egg-laying breeds in my care in a preventative capacity (i.e. my hens are routinely implanted to try and prevent health problems arising)*
- *Yes, for all egg-laying breeds in my care in a treatment capacity (i.e. my hens are only implanted if health problems connected to egg-laying arise)*
- *No*

- *Other*
- *Not applicable - I only care for roosters/cocks*

Do you have any concerns about the Suprelorin hormonal implant? Please explain.

Extra questions for those selecting 29b:

The Suprelorin hormonal implant is often recommended by exotic vets to prolong the duration and quality of life for laying hens, who are susceptible to reproductive illnesses due to the number of eggs they have been engineered to breed—around 300 per year vs around 12 per year in their ancestors. The implant works by preventing the release of the hormone responsible for triggering egg production. Its cost will vary according to the region you live in and size of the implant. However, a rough figure is US$140 (£100 or €116) for the large size, which will need to be re-administered approximately every 10 months. It can cause some initial side effects (e.g., chickens becoming more subdued and a considerable moult); the side effects are thought to generally wear off after a couple of weeks. Hens may also be calmer overall on implants. Considering this information, would you ever consider using this hormonal implant on your hens?

Yes
Yes, but only if it was more affordable
No
Unsure
N/a (if only keep roosters).

30. If you have a hen in your care who stops laying eggs, which of the following applies?

I rehome her
I kill her/have her killed
I continue to look after her
I never have any hens in my care
All my hens are hormonally implanted, so they do not lay eggs

31. Do you have experience of caring for any chickens with special needs or who are disabled (e.g. a blind chicken)?

Yes (please share an example or two) No

For those selecting 'no': Do you think you would be willing to care for a chicken who has special needs or is disabled (e.g., a blind chicken)? Please explain your answer.

32. Do your chickens have a full plumage?

Yes, mostly Sometimes Rarely/never

33. Do you have arrangements in place for the care of your chickens in the event of your unexpected death (e.g. the care of your chickens is discussed in your will or you have spoken to a charity or family members about it)?

Yes Partially No

34. Please tick the box that most closely reflects how much you agree or disagree with the following statements related to your chicken care-taking practices.

Statement	*Never*	*Rarely*	*Sometimes*	*Often*	*Always*
I worry about mychickens' health and wellbeing					
I find caring for chickens easy					
I find caring for chickens stressful					
I prefer natural deaths at home for chickens over euthanasia					
I conduct periodical health checks of my chickens					
I ensure my chickens have fresh material for dustbathing in					
I have a post- mortem carried outto determine the cause of death if the cause of death of one of my chickens is unknown					
I deworm the chickens OR send off faecal samples for testing at the respective recommended time intervals					
I let my chickens inside my house if they wish to come in					
I let my chickens rest for long periods inside the house if they wish to					
I take my chickens on trips (e.g., to thebeach or a woodland)					
I spend time observing my chickens' behaviour					
I use a chicken diaper on my chickens					
I arrange for someone to care for my chickens when I am away for a weekend					
I use a chicken sweater on my chickens					
I try to improve my knowledge of optimal care-taking practices for chickens					
I feed any eggs my chickens lay back to them					
I use a chicken leash					
I introduce new chickens to a flock gradually over a period of a few weeks					
I clean my chickens' hind feathers if they become very soiled					

I include a probiotic supplement in my chickens water/feed					
I include a calcium supplement in my chickens water/feed					
Regardless of the cost, if there is a treatment that could help my chicken, then I pay for it or fundraise for it if I can't afford it					
I find caring for chickens harder than I thought I would					
I am confident in providing excellent care for my chickens					

35. Can you think of any novel/niche chicken care-taking practices that you think warrant more attention and support? Please explain.

Section 3: Your relationship with your chicken(s)

36. Please tick the box that most closely reflects how much you agree or disagree with the following statements that are related to the relationship you have with your chicken (bird). Please consider the same chicken throughout this question, namely the one you are closest to.

Statement	*Strongly disagree*	*Disagree*	*Undecided*	*Agree*	*Strongly agree*
I enjoy playing with my bird					
I think my bird understands me					
My bird knows when I'm feeling bad					
I consider my bird to be a friend					
My bird is an equal member of my family					
Sometimes I wonder what my bird is thinking					
I can talk to my bird about anything					
My bird is like a child to me					
My bird provides structure for my life					
Having a bird gives me something to care for					
My bird makes me feel needed					
Spending time with my bird makes me forget my problems for a while					
I feel relaxed / more content because of my bird					
I feel distressed when my bird is ill and I see [my bird] suffering					
When my bird is ill, it is my duty to care for [him/her]					
I pay attention to my bird's body language					
My bird has [his/her] own unique personality					
My bird is a sensitive being with his/her own needs					
My bird actively tries to be close to me					
My bird always keeps a little distance from me					
My bird ignores me					

Section 4: Your attitudes towards chickens

37. Please tick the box that most closely reflects how much you agree or disagree with the following statements that are related to your attitudes towards chickens.

Statement	*Strongly disagree*	*Disagree*	*Undecided*	*Agree*	*Strongly agree*
It is morally wrong to participate in cockfighting in anyway					
It is morally acceptable to keep chickens for their eggs					
I think it is perfectly acceptable for chickens to be raised for human consumption					
Basically, humans have the right to use chickens as we see fit					
The slaughter of chickens should be immediately stopped even if it means some people will be put out of work					
I sometimes get upset when I see chickens in cages					
Breeding chickens for optimal egg production is morally acceptable					
Sometimes it is necessary to clip chickens' wings to prevent them jumping/flying too high					
It is morally wrong to breed chickens when millions of male chickens are unwanted and killed					
Chickens are morally less important than dogs					
Chickens experience a wide variety of emotions					
Chickens are unintelligent					

Section 5: The future

38. Do you see yourself still caring for chickens in 10 years' time?

Yes No Unsure

Please explain your answer.

39. How do you think the welfare of your chickens could be further optimised?

40. What better support do you think could be available to help you to care optimally for your chickens?

41. Is there anything else that you would like to share in relation to caring for your chickens?

42. Please enter your email address below if you would like to receive further information about this study and to be entered into the prize draw.

Section 6: Human demographics

43. Please confirm that you care for chickens in a non-commercial capacity (i.e., you do not make any money from them).

I care for chickens in a non-commercial capacity
I do not care for chickens in a non-commercial capacity

44. Please select your age group.

18-24 25-34 35-44 45-54 55-64 65-74 75+

45. Which of the following best describes the highest education level you have completed?

Lower secondary/high school
Upper secondary school/Sixth Form
Some higher education but not to degree level
Undergraduate degree
Master's degree
PhD

46. Please select the gender you identify most with:

Male Female Non-binary Rather not say

47. What is your ethnicity?

48. What country do you live in most of the time?

USA
Canada
Australia
New Zealand
UK
An EU country

Another European country (please state)
Other (please state)

49. Which of these options best describes your living environment?
Rural Semi-rural Suburban Urban

50. Which of these options best describes your home?
Terraced house Semi-detached house Detached house Other (please state)

51. Which of these applies to you?
I rent my home / I own my home

52. How would you describe the size of your garden/land?
Small Medium Large Very large

53. Which of the following best describes your occupation?
Professional
Clerical
Sales
Service
Labour (other than agriculture)
Agriculture
Student
Retired
Unemployed
Other

54. What is your household profile?
Single-person household
Multi-adult household (e.g., roommates or friends)
Married/living with partner with no children (do not have any)
Married/living with partner with no children (they live elsewhere/are grown-up)
Married/living with partner with children
Other

55. Which option do you think best describes your general diet?
- Standard omnivore (I eat animal products as part of most meals)
- Conscientious omnivore (I only purchase animal products from local and high-welfare sources)
- The only animal product I do NOT consume is chicken meat
- Reducetarian (I am reducing my consumption of animal products)
- Pescatarian (I do not eat meat, but I do eat fish and/or seafood)
- Pollotarian (I do not eat red meat, but I do eat white meat)
- Vegetarian (I do not eat meat, fish, or seafood, but I consume dairy and eggs or products containing these)

- The *only* animal products I consume are eggs from my hens
- Plant-based/vegan (I avoid consuming all animal products and aim for a 100% plant-based diet)
- Other (please state)

56. Are you responsible for the care of other animals in addition to chickens? You can select multiple answers if applicable.?

- No
- Yes, dog(s)
- Yes, cat(s)
- Yes, other (typically) outdoor birds such as ducks or turkeys
- Yes, other small animals such as guinea pigs, rabbits or hamsters
- Yes, other indoor birds such as parrots or budgies
- Yes, fish
- Yes, other exotic pets such as snakes, tortoises, tarantulas or lizards
- Yes, large animals such as horses, cows, pigs, sheep or goats
- Yes, another type of animal (please state)

57. How long have you been caring for chickens?

Less than 1 year
Between 1 year and under 3 years
Between 3 years and under 5 years
Between 5 years and under 7 years
Between 7 years and under 9 years
Between 9 years and under 11 years
More than 11 years

Many thanks indeed for taking the time to complete this questionnaire.

Supplementary Materials B. Supplementary figures

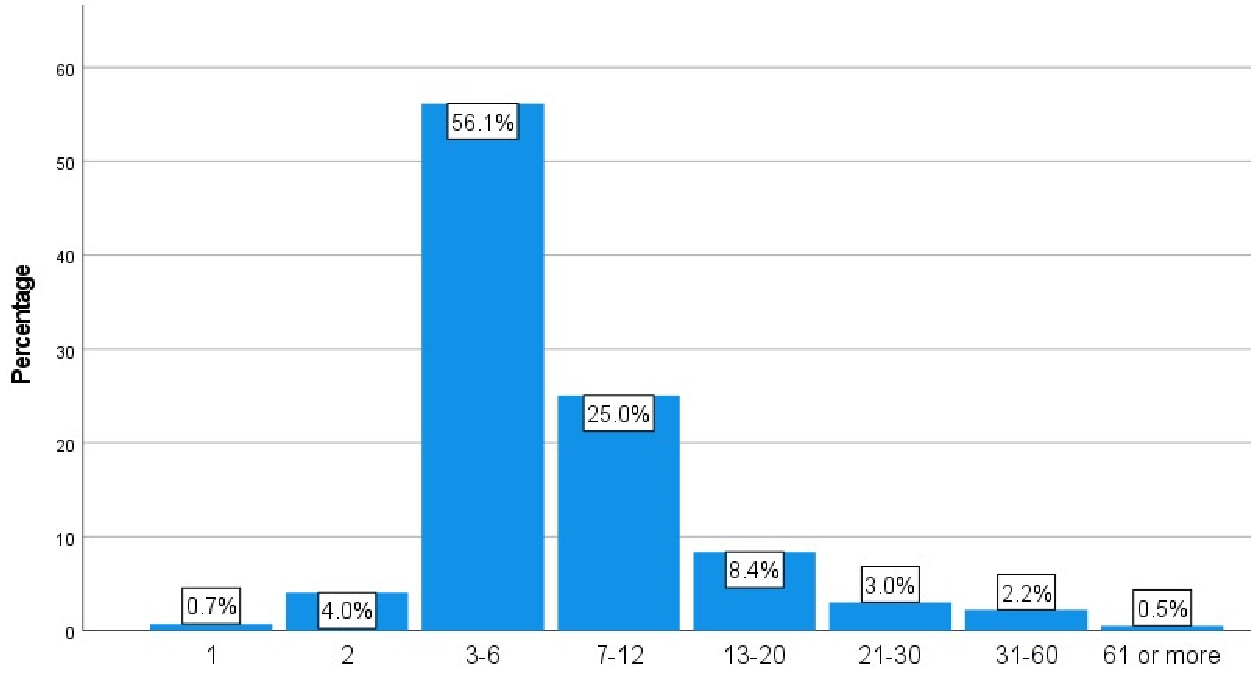

Figure S1. The number of chickens in respondents' care (n=2,058).

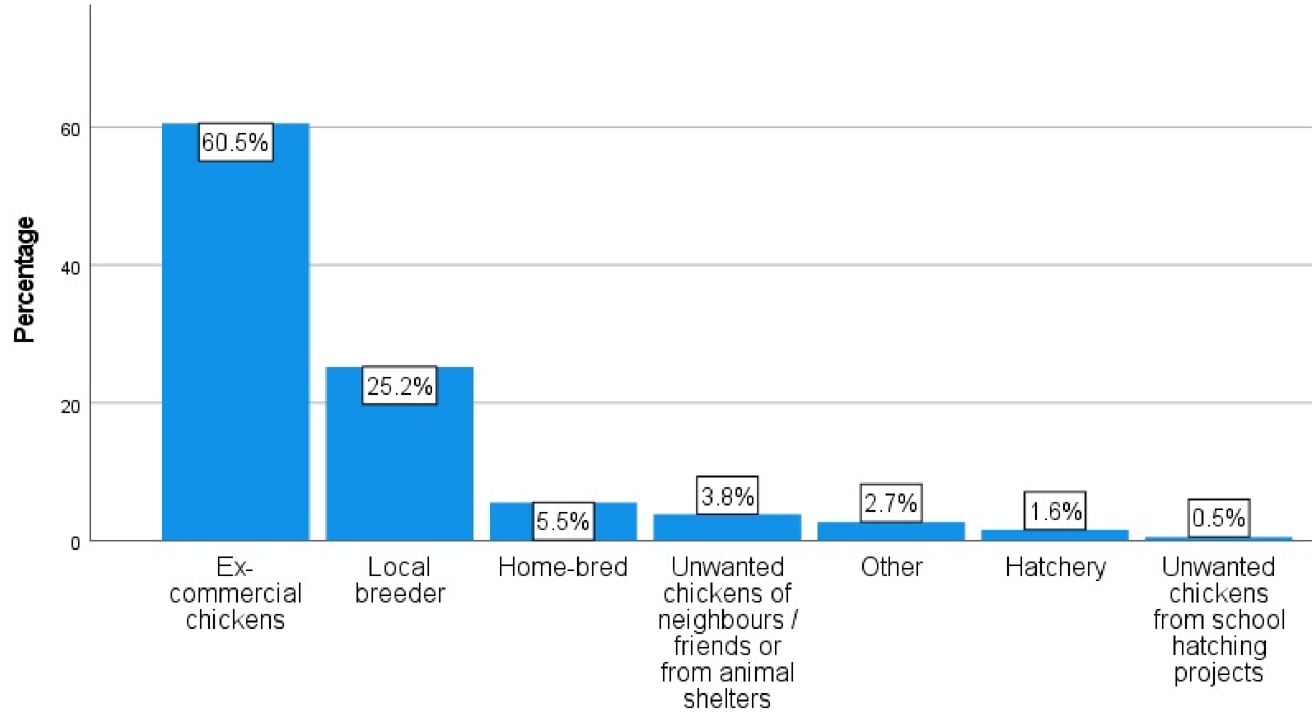

Figure S2. The origin of the majority of chickens in respondents' care (n=2,057).

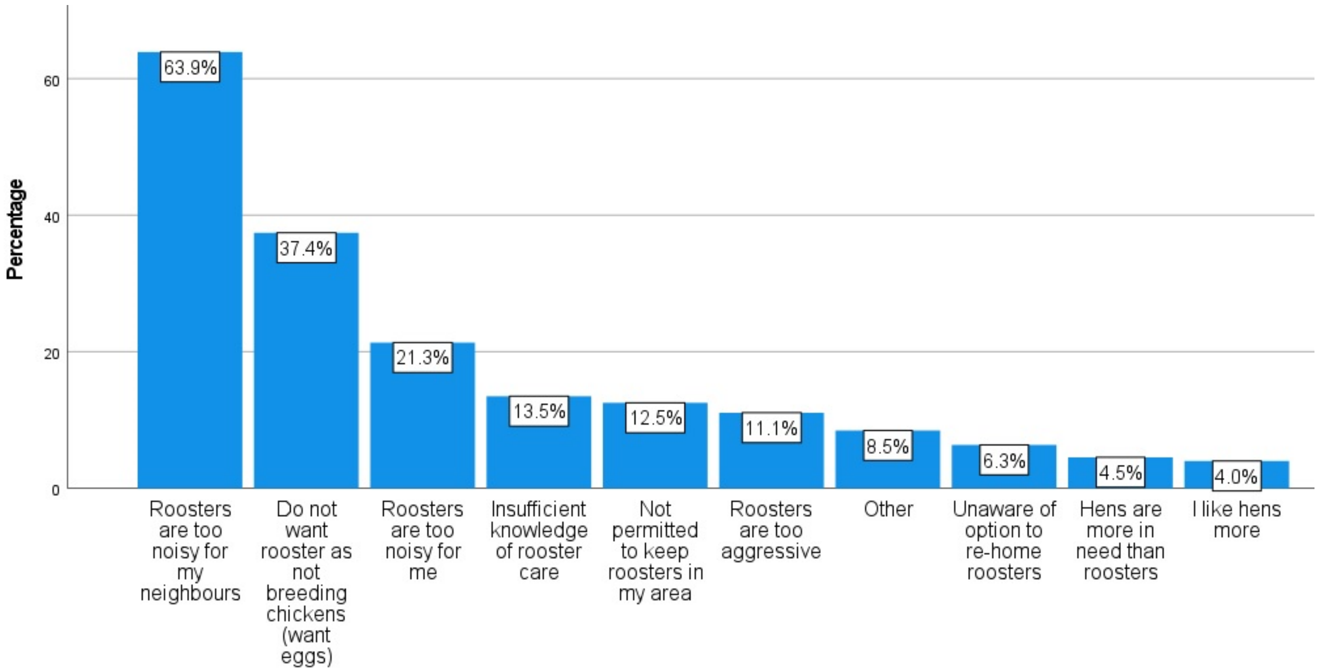

Figure S3. Respondents' reasons for caring solely for hens, and not roosters/cocks.

Note: Each answer choice was treated as its own independent dichotomous question ("yes"=reason was applicable; "no"=reason was not applicable); the percentages shown represent the percentage of respondents choosing "yes" for each answer choice. Respondents could select multiple answer choices, meaning the total number of respondents is not stated. This question was just for those who stated they did not care for any roosters.

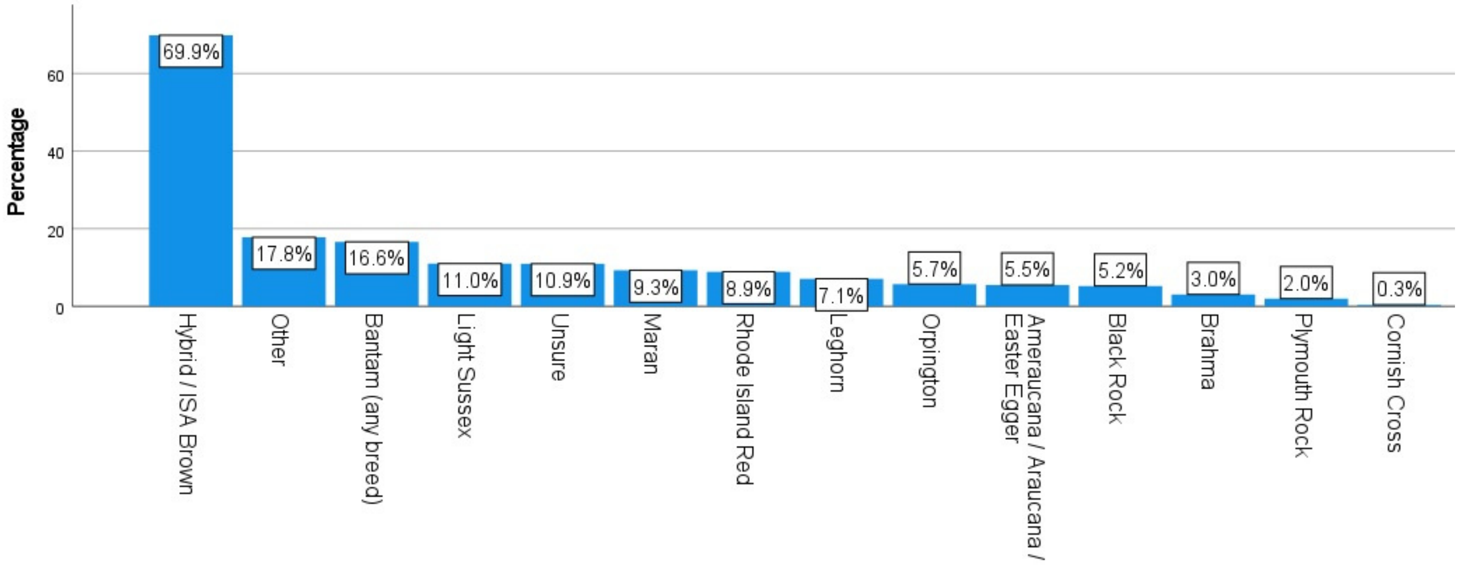

Figure S4. The most common breed/type of chicken cared for by respondents.

Note: Each answer choice was treated as its own independent dichotomous question ("yes"=breed/type was owned; "no"=breed/type was not owned); the percentages shown represent the percentage of respondents choosing "yes" for each answer choice. Respondents could select multiple answer choices, meaning the total number of respondents is not stated. Examples of "other" breeds included Nero, Bluebell, Australorp, Silkie, Welsummer, and Wyandotte.

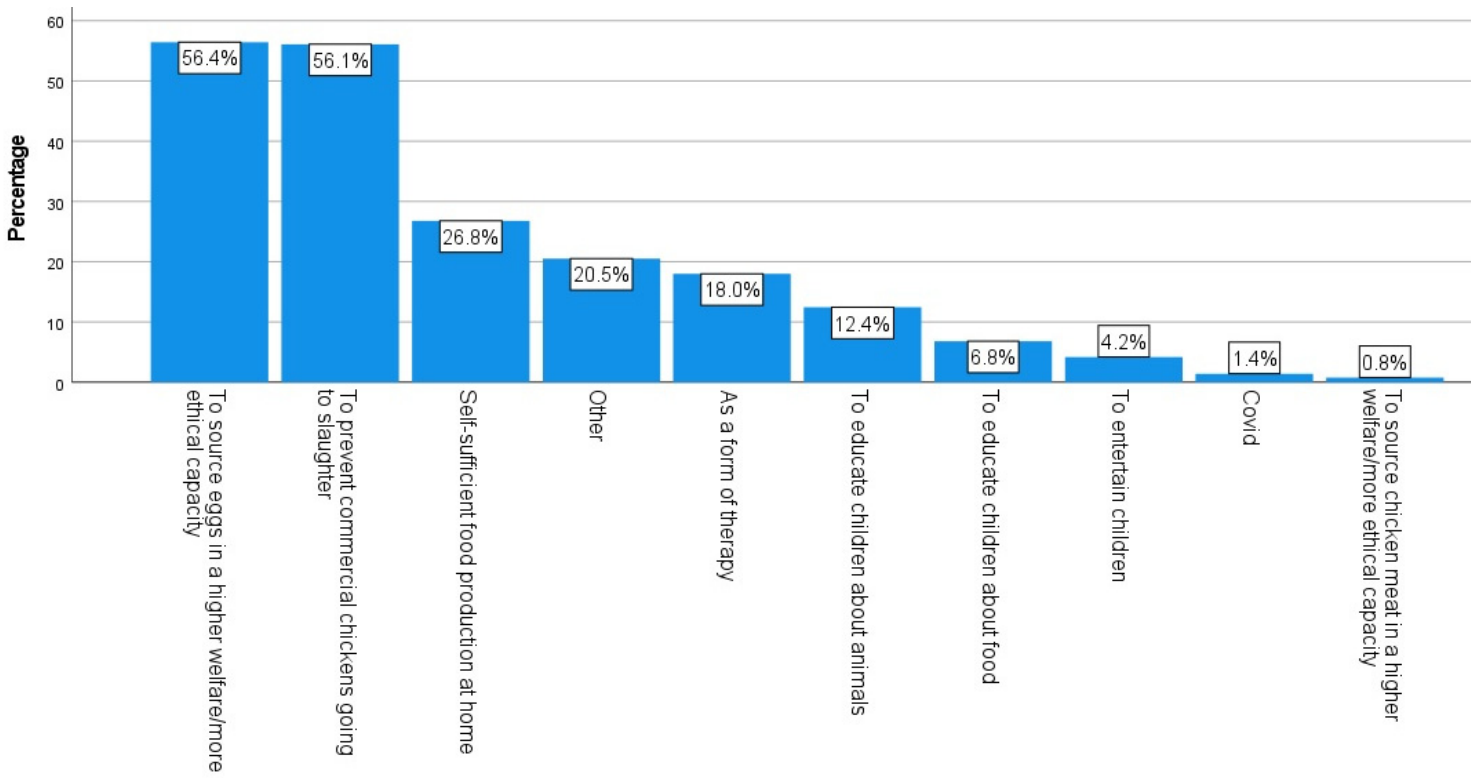

Figure S5. The primary motivations of respondents for caring for chickens.

Note: Each answer choice was treated as its own independent dichotomous question (“yes”=motive was applicable; “no”=motive was not applicable); the percentages shown represent the percentage of respondents choosing “yes” for each answer choice. Respondents could select multiple answer choices, meaning the total number of respondents is not stated. The main “other” motivation was for pets, as companion animals, and for enjoyment.

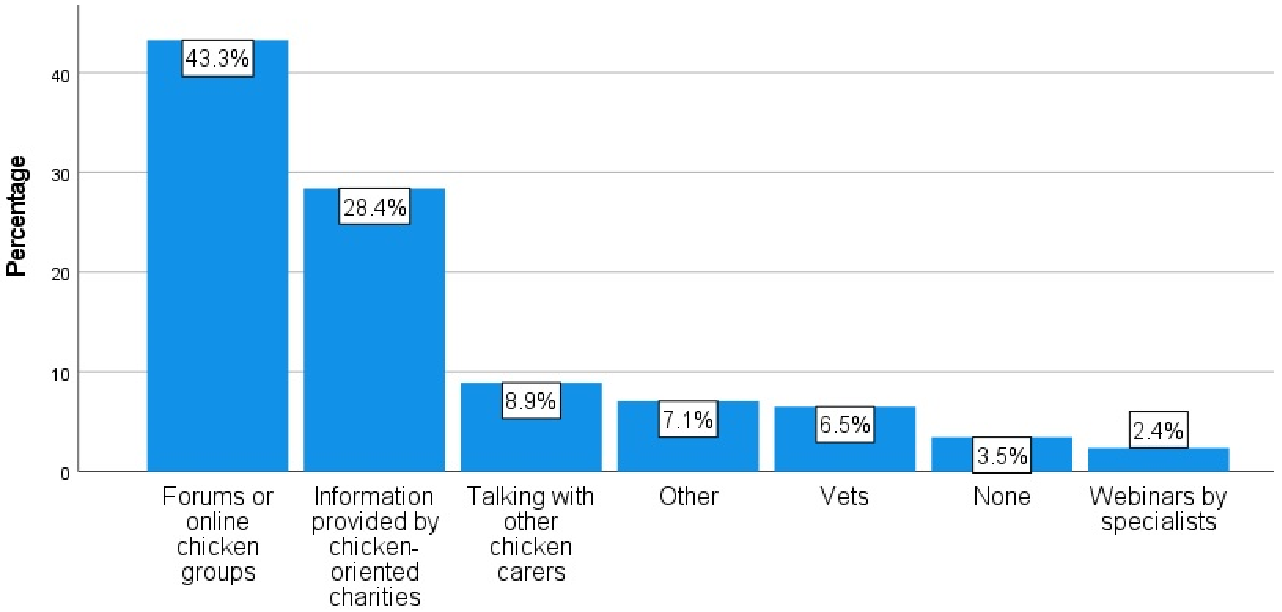

Figure S6. The care resource most commonly used by respondents (n=2,055).

Supplementary Materials C. Supplementary tables

Table S1. Additional care-taking practices.

	Never	Rarely	Some times	Often	Always
I worry about my chickens' health and wellbeing	1.3%	10.5%	45.9%	25.5%	16.9%
I find caring for chickens easy	0.3%	1.3%	14.0%	54.5%	29.9%
I find caring for chickens stressful	29.2%	41.7%	25.6%	2.8%	0.6%
I take my chickens on trips (e.g., to the beach or a woodland)	94.5%	3.3%	1.2%	0.7%	0.3%
I use a chicken diaper on my chickens	97.5%	1.4%	0.8%	0.2%	0.1%
I use a chicken sweater on my chickens	91.6%	4.6%	2.9%	0.6%	0.3%
I use a chicken leash	98.3%	1.0%	0.2%	0.0%	0.4%
I include a calcium supplement in my chickens water/feed	22.7%	9.2%	26.9%	20.9%	20.3%
I find caring for chickens harder than I thought I would	44.3%	24.7%	19.9%	7.0%	4.1%
I am confident that I provide excellent care for my chickens	1.1%	1.6%	7.8%	41.2%	48.4%

Table S2. Full chi-squared test of association results.

Note: Full results of the chi-squared tests of association between care-taking variables and demographic variables. Any significance highlighted by the chi-squared tests are highlighted in bold with the Cramer's V strength of association value added (0 = no association; 0.1-0.2, weak association; 0.3 = moderate association; over 0.3 = strong association, as per the Methods section). Cramer's V values 0.2+ are in red. In instances where cell counts were <20% in five+ cases, the likelihood ratio test was used in lieu of the chi-squared test; such cases are highlighted in grey.

	**Demographic variables**										
**Carer type/ style**	**Human age**	**Gender**	**Country**	**Home type**	**Occup-ation**	**House-hold profile**	**Living env**	**Educ-ation level**	**Diet**	**Diet group**	**Role name**
Source most	**0.028, .065**	0.494	**<0.001, .203**	0.09	**0.003, .099**	**0.007, .072**	**<0.001, .093**	0.238	**<0.001 , .117**	**<0.001, .109**	**<0.001, .214**
Hens, cocks, both	0.840	0.140	**<0.001, .272**	**<0.001, .109**	**0.032, .109**	0.624	**<0.001, .157**	0.224	0.440	0.118	**0.01, .08**
Name chickens?	**<0.001, .184**	**<0.001, .107**	**0.011, .073**	**<0.001, .082**	**<0.001, .153**	**<0.001, .143**	**<0.001, .122**	0.054	**0.001, .100**	**<0.001, .075**	**<0.001, .109**
Setup	**<0.001, .086**	0.314	0.165	0.055	**0.015, .101**	**0.004, .074**	**<0.001, .135**	**0.03, .071**	0.06	0.063	0.131
Coop material	**<0.001, .108**	0.207	**0.006, .087**	**0.010, .071**	**<0.001, .104**	**0.010, .076**	**<0.001, .101**	0.301	0.067	0.128	0.102
Memori-alize?	**<0.001, .199**	0.057	**0.003, .095**	**<0.001, .092**	**<0.001, .157**	**<0.001, .147**	**0.005, .069**	0.687	**<0.001 , .190**	**<0.001, .139**	**<0.001, .155**
Take to vets?	**<0.001, .120**	**<0.001, .076**	**<0.001, .105**	0.885	**<0.001, .111**	**<0.001, .086**	**<0.001, .077**	**0.014 , .070**	**<0.001 , .127**	**<0.001, .092**	**<0.001, .125**
Home treatment success?	**0.014, .146**	0.800	0.198	0.280	0.060	0.57	0.415	0.306	0.923	0.896	0.280

Happy with vets?	0.252	0.445	0.059	**<0.001, .106**	0.602	0.688	0.297	**0.011 , .095**	0.067	0.266	0.351
Consider implant?	**<0.001, .135**	0.134	0.187	**0.016, .078**	**<0.001, .135**	**0.003, .091**	**0.049, .064**	0.707	**0.002, .121**	**<0.001, .094**	**<0.001, .111**
Implants improve lifespan	0.083	.179	0.363	**0.047, .106**	0.296	.237	**0.029, .103**	0.7	**<0.001 , .193**	**0.001, .127**	0.202
Implants improve life quality	**0.014, .140**	0.373	0.119	0.632	**0.014, .179**	0.391	0.312	0.981	**0.008, .157**	**0.002, .115**	0.314
Regularly use implants	**0.031, .120**	**0.009, .227**	0.213	**0.037, .110**	0.117	**0.011, .138**	0.249	0.165	**<0.001 , .209**	**<0.001, .198**	0.053
Approach if stop laying	0.064	0.334	0.211	0.816	0.087	0.387	**0.038, .066**	0.367	**<0.001 , .128**	**<0.001, .137**	**<0.001, .081**
In will?	**<0.001, .106**	**0.043, .054**	**0.001, .088**	0.074	0.108	**<0.001, .113**	**<0.001, .078**	**0.001 , .084**	**<0.001 , .115**	**0.001, .074**	**<0.001, .128**
Daily feces removal from run?	**0.015, .104**	0.352	0.360	0.766	0.217	**0.005, .107**	0.358	**<0.00 1, .142**	**<0.001 , .145**	**0.005, .094**	**<0.001, .166**
Heard of implant?	**<0.001, .211**	**<0.001, .120**	0.175	0.562	**0.003, .110**	**<0.001, .166**	0.396	0.111	**<0.001 , .222**	**<0.001, .190**	**<0.001, .100**
Cared for special needs?	**0.005, .096**	**0.007, .077**	**<0.001, .170**	0.381	0.09	0.076	0.878	0.226	**<0.001 , .188**	**<0.001, .162**	**<0.001, .158**
Able to select closest chicken?	**<0.001, .187**	**<0.001, .110**	**0.018, .083**	0.244	**<0.001, .132**	**0.003, .095**	0.933	0.338	**<0.001 , .148**	**<0.001, .113**	**<0.001, .174**
No. of chickens	0.180	0.936	**<0.001, .115**	**<0.001, .120**	**0.003, .093**	0.179	**<0.001, .166**	**0.013 , .073**	0.069	0.288	**0.006, .091**
How long caring	<0.001, .171	0.032, .069	0.398	0.014, .071	<0.001, .115	<0.001, .094	<0.001, .108	0.926	0.153	0.635	0.017, .071
Stage of life	0.002, .078	0.576	<0.001, .174	0.017, .064	<0.001, .098	<0.001, .076	<0.001, .075	0.026 , .065	<0.001 , .120	<0.001, .089	<0.001, .207
Frequency coop feces removal	0.017, .067	0.117	<0.001, .145	0.041, .073	0.726	<0.001, .083	0.159	0.074	<0.001 , .101	<0.001, .098	<0.001, .110
Run size	0.001, .0.089	0.147	0.408	<0.001, .106	<0.001, .106	<0.001, .099	<0.001, .182	0.367	0.381	0.672	0.163

Table S3. Contingency table comparing method of acquiring chickens and country of residence.

	What country do you live in most of the time?								
	**USA**	**Canada**	**Austra-lia**	**New Zealand**	**UK**	**An EU country**	**Another European country**	**Other**	**Total**
The chickens in my care came from a hatchery	14	0	0	0	17	1	0	0	32
The chickens in my care came from a local breeder	6	1	1	1	494	8	1	3	515
I bred and reared the chickens in my care	4	0	2	0	99	6	0	2	113
The chickens in my care are ex-commercial chickens spared from slaughter	6	0	0	1	1222	6	1	4	1240
The chickens in my care are unwanted chickens from school hatching projects	0	0	0	0	10	0	1	0	11
The chickens in my care are unwanted chickens of neighbors or friends or from animal shelters	12	0	0	0	64	2	0	0	78
Other	4	2	0	0	48	1	0	1	56
Total	46	3	3	2	1954	24	3	10	2045

Table S4. Contingency table comparing method of acquiring chickens with role name.

	Which of the following terms do you think best describes your chicken-caring role?					
	Chicken rescuer	Chicken carer	Chicken keeper	Backyard farmer	Other	**Total**
The chickens in my care came from a hatchery	0	10	16	5	2	33
The chickens in my care came from a local breeder	4	195	282	27	10	518
I bred and reared the chickens in my care	3	30	67	7	7	114
The chickens in my care are ex-commercial chickens spared from slaughter	408	457	337	21	19	1242
The chickens in my care are unwanted chickens from school hatching projects	5	2	4	0	0	11
The chickens in my care are unwanted chickens of neighbors or friends or from animal shelters	13	37	25	2	1	78
Other	2	20	30	1	3	56
Total	435	751	761	63	42	2052

Table S5. Contingency table comparing specific chicken sex cared for with country of residence.

	What country do you live in most of the time?								
	**USA**	**Canada**	**Australia**	**New Zealand**	**UK**	**An EU country**	**Another European country**	**Other**	**Total**
I care for hens	17	1	1	2	1604	13	1	6	1645
I care for roosters/cocks	0	0	0	0	2	0	1	0	3
I care for both hens and roosters/cocks	28	1	2	0	342	10	1	4	388
Total	45	2	3	2	1948	23	3	10	2036

Table S6. Contingency table comparing use of implants with gender.

	Please select the gender you identify most with				Total
	**Female**	**Male**	**Non-binary**	**Rather not say**	**Total**
Use implants for all hens in a preventative capacity	11	3	0	0	14
Use implants for all hens in a treatment capacity (only if become ill)	100	13	1	0	114
No	467	23	0	7	497
Other	26	1	0	0	27
Not applicable - I only care for roosters/cocks	0	0	0	1	1
Total	604	40	1	8	653

Table S7. Contingency table comparing use of implants with diet group.

	Diet Grouped				Total
	Omni -vore	Vege- tarian	Plant- based/vegan	Other	
Use implants for all hens in a preventative capacity	4	1	9	0	14
Use implants for all hens in a treatment capacity (only if become ill)	68	30	14	2	114
No	349	107	32	8	496
Other	12	7	7	1	27
Not applicable - I only care for roosters/cocks	0	0	1	0	1
Total	433	145	63	11	652

Table S8. Contingency table comparing awareness of implant and age group.

		Please select your age group.							Total
		18-24	25-34	35-44	45-54	55-64	65-74	75+	
Have you heard of the Suprelorin hormonal implant that can be used in hens?	Yes	5	85	127	207	172	54	4	654
	No	10	71	175	411	444	241	38	1390
Total		15	156	302	618	616	295	42	2044

Table S9. Contingency table comparing awareness of implant with diet group.

		Diet Grouped				Total
		Omnivore	Vegetarian	Plant- based/vegan	Other	
Have you heard of the Suprelorin hormonal implant that can be used in hens?	Yes	435	145	63	11	654
	No	1125	214	34	15	1388
Total		1560	359	97	26	2042

Table S10. Novel care-taking practices.

Note: Tallies of qualitative categories of responses are provided regarding any novel care-taking practices respondents felt deserved more attention or research.

*The authors are merely reporting the respondents' comments. The authors recommend consultation with an exotic/avian vet before attempting any novel treatment with chickens. There is no evidence (beyond anecdotal) of the safety of this treatment option and the authors do not recommend its use.

**This is illegal in the UK though legal in many countries such as across the EU [76].

Category	Subcategory	Tally	Total
Home remedies	Adding apple cider vinegar and garlic to water	20	41
	Herbal remedies to avoid medications (e.g., St John's Wort, oregano oil, turmeric, kefir, seaweed)	13	
	Aspirin for circulation or pain relief*	3	
	Brandy to treat sour crop*	2	
	Wine (for improved eggs)*	1	
	Cannabis oil	1	
	Nystatin cream for vent gleet	1	
Other	More space/enrichment (music/radio, hanging veg, bottles with holes for corn pecking, particular concern for free-range ex-commercial hens being kept in small runs, more shrubs in run).	28	119
	Interact more with chickens/chicken training	28	
	Red mite avoidance (optimizing use of DE, Elanco/Elector, (unpermitted) use of Frontline intended for dogs/cats*)	23	
	Feed eggs back to them	5	
	Hen integration (keep separate for weeks, then introduce at night)	5	
	Use of vets	3	
	Chicken trolley, wheelchair, hammock, saddle	3	
	Bathe chickens	3	
	Regular poo picking of run	2	
	Do worm counts vs deworming	2	
	Treadle feeders	2	
	DIY crop feeding to ill chickens	2	
	Deep litter method	2	
	Use heat in coop	2	
	Chicken massage/physio	1	
	Treat crop issues with digestive enzyme	1	
	Rooster chemical caponization**	1	
	Take on trips/use leash	1	
	Llamas as lookout animals	1	
	Create soft landing areas from perches for heavier chickens (joints)	1	
	2-run system and alternate to reduce disease	1	
	Chicken insurance	1	
	Conduct health checks	1	

Table S11. How can non-commercial chicken welfare be further optimized?

Note: Categories of qualitative responses are provided regarding how non-commercial chicken welfare could be further optimized (n=1,644). Respondents frequently stated multiple factors; hence the total responses exceed the number of respondents.

**Category**	**Subcategory**	**Tally**	**Total**
Vets care	Better vet care	109	232
	Local avian vet	58	
	Reduce cost of vets/funding for costs/subsidized costs	32	
	Funding for implants	14	
	Better vet ethics	7	
	Mobile avian vet	6	
	More meds licensed for chickens	3	
	Over-the-counter meds for chickens	3	
Living quarters	More space (coop, run, free-range time)	511	921
	Effective predator protection/deterrent for secure large area	193	
	Environmental enrichment	52	
	Improved equipment	50	
	More grass/plants/greenery	40	
	More shelter (shade, rain)	31	
	More varied space	26	
	Use plastic coop	10	
	Closer to home	3	
	Sun in winter	2	
	Better run substrate	1	
	Less mud	1	
	Persuade 12 rescued together to not sleep in same coop	1	
Care	Spend more time, give more attention/energy	40	161
	Prepare for future avian flu lockdowns	22	
	Climate control (cool in Summer and warm in winter)	21	
	More regular cleaning	20	
	Improve diet (e.g., organic)	12	
	Give probiotics/supplements	10	
	Improve worming consistency/do count/make easier	7	
	Allow chickens inside human home more	7	
	More treats	5	
	Let out earlier in Summer/use automatic door	5	
	More health checks	5	
	Teach/train	3	
	Don’t allow inside human home	2	
	Trips to new areas	1	
	Difficulty identifying “problem” hen	1	
General	Rescuing/having more chickens	19	52
	Eliminate red mite/parasites	14	
	Rat control	8	
	Few cats stalking chickens	4	
	No bullying/fighting	4	
	Rescuing/having fewer chickens	2	

	Tamer chickens	1	
Broader society	Acquire more knowledge/training courses/education of owners	102	266
	Ban intensive farming in cages and barns	52	
	Funding for setup	18	
	Less breeding for maximum egg production	15	
	More awareness of ex-commercial hen health issues, vet costs, and space requirements	12	
	Attitudes/legislation to shift so status similar to cats/dogs	12	
	Permit roosters	12	
	Stop eating chickens and hens' eggs	7	
	Increase public awareness about welfare costs of cheap meat/eggs	7	
	Stop chicken lockdown/develop vaccine	6	
	Chicken pet insurance	5	
	Defra - change attitude towards backyard carers: "we're not farmers"	4	
	Welfare inspections / state some BY practices as illegal	3	
	More chicken sitters	3	
	Improve representation on TV/in pet shops	2	
	Ban school hatching projects	1	
	Promote capon	1	
	Sex-testing to avoid males	1	
	Compulsory registration	1	
	Runs/coops on the market are too small for stated number	1	
	Fewer neighbor worries in terms of noise	1	
Other	Believes already have high (enough) welfare: nothing further needed	209	209

Table S12. What support would aid chicken welfare?

Note: Categories of qualitative responses are provided regarding what support would aid the enhanced welfare discussed in Table C11.

**Category**	**Subcategory**	**Tally**	**Total**
Vets	Knowledgeable avian vets available locally	501	663
	Cheaper vets	85	
	Vets' attitudes to chickens to be similar to cats and dog	23	
	Online/phone vet consultations	20	
	Mobile avian vet	12	
	More licensed drugs for chickens	8	
	Access to vaccines for small flocks	6	
	Lower cost of implant	6	
	24/7 avian vet	1	
	Easier access to post-mortems	1	
Other specialist support	Local specialists/experienced carers – courses, workshops, network	94	289
	More specialist online resources	47	
	More official literature and non-contradicting information	34	
	More official research into optimal chicken welfare/health	25	
	More experienced chicken sitters/hotels/respite carers	21	
	More information at the start	13	
	Pet chicken insurance	9	
	Tailored support for rescued/disabled chickens	8	
	Helpline	6	
	Wider awareness of implants	5	
	Inspections/welfare visits/vetting	5	
	More on rooster care	3	
	Euthanasia method without vets	3	
	Research into natural/home remedies	3	
	Vegan care resources	3	
	More discussion of chicken deaths	3	
	Wider awareness of chicken diaper and mat-training chickens	2	
	Campaigns to prevent certain practices such as bathing chickens and tipping chickens upside down to remove crop liquid	2	
	Improved Defra bird flu updates	1	
	More effective hormone blocker	1	
	Research regarding selecting for quieter rooster crow or from later time	1	
General	More time/money	31	150
	Improved predator deterrents/protection	17	
	Wider availability of chicken feed and equipment	15	
	Educating the public about chicken capacities/sentience/role as pets	12	
	Council support	11	
	Over-the-counter meds	10	
	Help from others (family/friends)	8	
	Subsidies for rescued chickens	7	
	Better chicken products on the market	7	
	More enrichment ideas	6	
	More effort from me	6	
	End all anti-chicken zoning laws	5	

	Encourage urban chicken keeping	2	
	Legal recognition of chickens as pets vs farmed animals	2	
	Ban intensive farming/breeding for maximum egg yield	2	
	Better carer health	2	
	More diverse feed for chickens other than just pellets	2	
	Climate-control in coops	1	
	Chicken-specific rescue centers akin to Battersea	1	
	No avian flu lockdown	1	
	Humane rat control	1	
	Compulsory registration	1	
Other	Already sufficient support available	60	X
	Same as previous Q	26	
	Not sure	126	
	None – don't need/want help	192	

Table S13. Qualitative responses to "any other comments."

Category	Subcategory	Tally	Total
Chickens' role	Pleasure/rewarding/satisfying	139	280
	They are wonderful! / I love them!	53	
	Great pets / more recognition as pets	35	
	Great therapy	24	
	Useful	12	
	Part of family	8	
	Livestock, not pets	4	
	Not playthings	3	
	A kind of subpet	2	
Care-taking thoughts	Massive expense/commitment	50	89
	It is stressful/sad when they are ill/die	21	
	Easy	11	
	Introducing new hens is traumatic	3	
	Red mite is a nightmare	2	
	Different ways to care for birds	2	
Chicken characteristics	Unique/best personalities	43	108
	Friendly/intelligent/sentient	42	
	Interactive/show affection in own way	23	
Health	Improved vet care	29	39
	Love cuddle/lap time	7	
	Vets ASAP after going quiet	3	
Carer recommendations	Watch and spend time with them	21	107
	Encourage everyone to rescue ex-commercial chickens	18	
	Read as much as you can	12	
	Allow laying breaks	9	
	Enrichment	9	
	Ensure have shelter – shade and for storms	7	
	Terrible for garden	6	
	Great for garden	5	
	De-poop run regularly	3	
	BHWT resources	3	
	PoultryForum	3	
	Handy Hen Facebook group	2	
	Natural environments	2	
	Ensure none of your garden plants will kill them	2	
	Paddling pool in heat	1	
	Wood ash to bedding (red mite)	1	
	Clear up feed spills	1	
	Fencing type to avoid flying and landing on it	1	
	Avoid named veterinary practice	1	
	Small spaces should be discouraged – space to roam is required	43	
	More education required regarding chickens' needs at all life stages and of their benefits to humans	16	
	Encourage more to do it to reduce commercial demand	13	
	Increase awareness of commercial welfare abuses	12	
	Improvements re fox protection	11	

Societal recommendations	Greater tolerance of roosters	10	168
	Legislation to protect their rights from human harm	9	
	Rehoming charities could give a better start-up care package and vet adopters	8	
	More proactive council support	7	
	Chicken welfare carers' own responsibility	6	
	Stop breeding for maximum egg yield	4	
	Separate legislation to commercial chickens	4	
	Backyard chickens contribute to poor welfare too	4	
	Compulsory Defra registration	4	
	Chicken licenses and inspections	3	
	Ban school hatching projects	3	
	Breeders should be regulated just like cat/dog breeders	2	
	More advice required	2	
	Make vaccines available for small flocks	2	
	More chicken sitters	2	
	Track where end up	1	
	Cheaper materials	1	
	Relax renting rules	1	
Information	Spend time undoing harm from farm life	18	42
	Rescues have greater needs than POL hens	5	
	Closer with neighbors	5	
	Conflict with neighbors	4	
	Roosters help hens	3	
	So much more information available with onset of Internet than 40 years ago	2	
	Roosters disadvantage hens	1	
	Chickens mourn their mates	1	
	Can take years to get the right setup	1	
	Had influence on neighbors' egg-buying habits	1	
	Better relationship with rescues	1	
Children	Great for child education – animal welfare/husbandry	9	11
	Can be intimidating for young kids	2	
Concerns	New owners should do more research before adopting/acquiring	21	37
	Forum content	6	
	Rats	4	
	Allotment concerns (distance)	1	
	Appeared less happy in lockdown	1	
	Unhappy chickens in lockdown minor consideration vs human lives	1	
	Brexit, pandemic, food shortage leading to increase of thoughtless owners	1	
	Cannibalism if given cat food?	1	
	Chicken jumper bad	1	
Miscellaneous	Other	9	45
	Thank you for survey	7	
	Give them a life worth living	5	
	Dislike "backyard" term	4	
	Don't anthropomorphize	4	

Worry about feed sustainability	3
Addictive	2
Wish could move elsewhere to do more	2
Not meant to be vegetarian	2
Sounds liberal agenda/don't turn political/strange questions	2
Seemed less complicated 30 years ago	1
Ours are like in Chicken Run	1
I volunteer	1
Lone chicken attached to dog/toy	1
Don't understand lockdown logic	1
N/A or No	241

Supplementary Materials D. Discussion of results that supplement extant literature

Demographics

In terms of demographics, there seems to be relative consistency in non-commercial chicken carers' demographics. For instance, the most common flock size in this study of 3-6 chickens echoes averages of 3-5 found by Karabozhilova et al. [21] and Souvestre et al. [18], and under 10 by Elkhoraibi et al. [24]. Female chicken carers also vastly outweigh males: 86.9% versus 11.8% in this study, which mirrors similar imbalances in studies by Karabozhilova et al. [21], Elkhoraibi et al. [24], and Souvestre et al. [18]. Non-commercial chicken carers tended to live rurally in detached homes with large gardens, though this is diversifying, echoing the findings of Elkhoraibi et al. [24]. Middle-aged, married (with or without children) and high education levels were common demographic factors in this study and echoed by Elkhoraibi et al. [24], though this may be diversifying as well because Souvestre et al. [18] found 30-49 to be the most highly represented age group and an urban-based survey found the most common age group to be 25-40 [21].

Similar to other surveys, this survey had a low response rate from ethnic minorities. Whilst this can be partially explained by lower numbers of non-white ethnicities in the UK (the predominant country of residence for the vast majority of respondents in this survey), it may also be partially explained by the resources required to participate in surveys (e.g., time, internet, laptop, English skills), cultural norms, and a lower proportion of ethnic minorities living in rural areas, where non-commercial chicken keeping still dominates, despite diversification into non-rural areas. More research is warranted regarding chicken keeping in ethnic minority circles. Rehoming charities could also diversify their promotions to aid representation of ethnic minorities.

Chickens as pets and vet care for chickens

There are two key findings from this study that reinforce knowledge from extant literature: 1) the increasing number of non-commercial chicken carers who view their chickens as unkillable/inedible/a pet, and 2) a need for more avian-experienced and affordable vet care. These shall now be addressed in turn.

Firstly, 93.6% of respondents stated they did not, and would never, kill their chickens for human consumption (nor have someone do it for them). Moreover, 68.8% believed it to be morally unacceptable to breed chickens for maximum egg production, and over three quarters (76.3%) did not consider chickens to be morally less important than dogs. These are all traits commonly associated with pets/companion animals and with being higher on the sociozoologic ladder described by Arluke and Sanders [29]. When considering 68.8% of respondents finding it unacceptable to breed chickens for maximum egg production, there is comparable support amongst the public for bans on selective breeding with dogs who experience considerable health issues [77]. Moreover, whilst of the 10 possible motives provided, pet/companionship was not one of the motives offered in this study, the primary "other" options given in response to this motive question were "pet" or "companion." These results mirror high percentages of

chicken owners from other studies who view their chickens as pets: 53% and 57% in the studies by Souvestre et al. [18] and Elkhoraibi et al. [24], respectively, and 72% and 73% in the studies by Pohjola et al. [25] and Karabozhilova et al. [21], respectively. Some of the wording differed in the questionnaires such as that by Pohjola et al. [25] including "hobby, companion, pet" together as one motivation. This may explain the different ranges. More consistent wording in future survey research is required for more fruitful comparisons. Additionally, sample sizes were 30 [21], 178 [25], 1,160 [18], and 1,487 [24]; this variation may likewise explain some of the wide ranges in results. See section 4.3 for discussion of chicken rescue as a motive.

Secondly, the present study found 36.2% of all respondents did not use a vet. Of these, almost three quarters (74.0%) did attempt to treat their chickens at home though, and the top reason for this was a fear that vets would not know how to treat their chicken or just suggest euthanasia (selected by 55.1% of home treaters). The second most common reason selected was vets are too expensive (chosen by 45.3% of home treaters). Moreover, "improved vet care" was the third most commonly reported qualitative answer when respondents were asked how welfare could be further optimized and "knowledgeable avian vets available locally" was the top answer cited (n=501) when respondents were asked what could support them in optimizing welfare further. An urgent need for more avian knowledge amongst vets has also been reported by others such as Davies [40], Singleton et al. [19], and Kodilinye-Sims and Royden [78]. The fact that so many undiagnosed or non-specific symptoms have been identified in veterinary records for chickens provides further evidence for this lack of knowledge [27]. Similarly, in the present study, "unknown cause" was the most frequently selected answer option for cause of death, followed by "sudden death," suggesting there are big gaps in knowledge regarding chicken care and treatment. It would be useful to promote post-mortem services more to clients and for vets/universities to even subsidize the cost as post-mortems can help to advance knowledge. Akin to Elkhoraibi et al.'s [24] study that found only 8.8% had ever used necropsy services, in this study only 10.2% used such services either sometimes, often, or always.

Nevertheless, some potential improvements regarding veterinary care for chickens were also detected in the present study. For instance, the number of people *not* using a vet for chicken care may possibly be decreasing as the figure was up to 50% in Karabozhilova et al.'s [21] study (versus 36.2% in this study). To the authors' knowledge, the present study was the first survey-based study to distinguish between standard vet use and exotics vet use. This study found that 33.0% of those using a vet actually used an exotics vet. Whist this could be higher, for a baseline result, this is a significant proportion of chicken carers. Further distinctions could be considered in future studies such as adding in the option of a non-exotics but still less officially avian-experienced vet, as 47.7% of those using a non-exotics vet stated that this was the reason why they did not use an exotics vet (i.e., they used a 'chicken-friendly' vet or a vet with some level of avian experience). Another example of potential improvements in veterinary care for chickens is that almost three quarters (73.6%) of respondents in the present study reported they were happy with the health/welfare service received at their vets; only 7.5% reported they were unhappy, with a further 18.9% stating they were unsure. This could suggest that, generally speaking, those with good chicken vets available to them do make use

of them and are mostly happy with the service.

Further key welfare concerns

Alongside the lack of specialized veterinary care for chickens (discussed immediately above), there are some other key welfare concerns arising from this study that should be highlighted. These include: 1) higher reported incidences of and reasons for health concerns and deaths in the last year relative to extant literature, 2) welfare impacts arising from avian influenza lockdowns, 3) potentially inadequate spaces chickens are kept in, and 4) chicken killing methods – for the extreme minority that partake in killing their chickens. These will be addressed in turn.

First, 71.1% of respondents in the present study reported health conditions in their chickens in the last year, relative to only 41% in Elkhoraibi et al.'s [24] study – a 57.7% increase. Similarly, almost 70% (69.5%) reported 1+ deaths in the last year in the present study, relative to just 36% of respondents in Elkhoraibi et al.'s [24] study. Health condition prevalence and mortality in the last year were also reported as significantly lower in other studies too (e.g., [18]). The main reason for these apparent increases in health problems and deaths could be the high number of ex-commercial hens kept by the respondents in this study. This warrants further investigation. Ex-commercial chickens may require tailored care. It is potentially also an indictment of intensive animal farming – both living conditions and breeding regimens – if chickens adopted from such farms experience considerably worse welfare regarding regularity of health problems and likelihood of premature death.

This study reinforced external parasites (red mite) as the most common health condition, and found diarrhea among the top three health conditions reported, echoing Elkhoraibi et al. [24] and Pohjola et al. [25]. However, EYP emerged as the third most common health condition in the present study, which contrasts with former studies where prolapsed vent and sour crop were joint third. Nevertheless, Karabozhilova et al. [21] did also report an egg-related condition within the most commonly reported health conditions: egg binding. EYP reappears in our study as the fourth most common cause of death, preceded by unknown cause, sudden death and predation. In similar studies, cause of death has not commonly been reported in detail; however, predation was also a top cause of death in Elkhoraibi et al.'s [24] study.

Second, over 70% (71.0%) were worried about their chickens' welfare during the avian influenza "lockdown" restrictions in the winter of 2020-2021 in countries such as the UK (where the majority of respondents of the present study resided); 53.9% selected "yes" they were worried about the welfare of their chickens during avian flu restrictions, whilst 17.1% selected they were "sometimes" worried. In the open questions of this study, some respondents linked an increase in abnormal behavior – typically feather pecking – to the restrictions, even if owners had increased efforts with enrichment. As these restrictions are becoming more frequent [79], hopefully chicken carers are preparing better for further restrictions in future years, as some stated in the open questions of this study. However, for some it seems too resource-intensive in terms of time and cost. It should be made clear to prospective owners/rescuers that, come winter, these precautions will likely require adherence. As chickens descended from red junglefowl with roaming behavior as a highly

motivated behavior, it may be difficult for many (especially in urban areas) to cater for this need adequately under avian flu restrictions. This can lead to abnormal behaviors such as injurious pecking due to frustration and stress [80]. Other reasons for feather pecking could be inadequate procedures for introducing new chickens to a flock. In this study, over 30% stated they do not always introduce new hens gradually. Best practice is to keep new chickens separate but visible and introduce them slowly after several weeks of separation [81]. Some respondents also echoed concerns reported elsewhere [78] regarding some vets' refusals to treat chickens during avian influenza restrictions.

Third, there are several reasons to believe that many chickens at home may be kept in inadequate outdoor spaces (for indoor spaces, see section 4.3). These include: a) "more space" was mentioned most commonly (n=501) by those answering the qualitative question about how welfare could be further optimized; b) predation was the third most commonly reported cause of death (selected by 25.4%); c) few of the steps listed to minimize predation were selected by over 50% of respondents as being practiced; d) 34.1% stated they either only allow supervised free-range time (26.7%) or they never allow it at all (7.4%); and e) 56.0% of respondents deem temperatures -5°C (32°F) or colder as suitable as a minimum regular temperature for chickens. Hence, for a mixture of reasons surrounding enrichment of chickens' lives (providing positive behavioral opportunities), reducing behavioral problems, eradicating or minimizing predation risk, and keeping chickens at a comfortable temperature, chicken carers need to consider chickens' space requirements carefully. This is particularly concerning for chickens who may be rehomed either from an organic/genuinely free-ranging farm or a free-ranging home setup to confinement – even to a large run.

There is an argument to be made that the welfare enhancements to come from allowing chickens at home to free-roam outweigh the predation risk (in lower-risk areas), and that even if their lives are cut short, they have accrued so much welfare benefit, that it is justifiable to free-roam them – comparable to debates about cat free-roaming safety regarding potential car accidents. Nevertheless, the results of this study suggest there is considerable room for improvement in terms of minimizing predation risk. However, the third most commonly cited (n=50) response in the general open comments at the end of the study was the massive expense and time commitment that chicken care entails – often beyond people's expectations. Thus, these aspects should be emphasized to potential new carers more to avoid chickens being acquired on a whim and under false pretenses of caring for them being easy (indeed, only 29.9% selected "always" to the statement they are easy).

Also related to space issues, there is potentially a welfare risk from the use of just bare earth (selected by 41.1%) as the run substrate. This can lead to problems with mud during periods of high rainfall, especially in the winter. This can lead to increased disease risks so owners should be encouraged to add hard woodchips or another safe substrate to run floors. Finally, in this study, there was a 50:50 split between respondents removing chicken feces from the run on a daily basis, and those not doing so. Feces also increase disease risks; thus, owners should be encouraged to remove feces regularly.

Fourthly, a considerable 6.4% stated they (or someone on their behalf) kill their chickens for

human consumption. This is roughly half the percentage of respondents who stated they killed their chickens for meat in Elkoraibi et al.'s [24] and Correia-Gomes and Sparks' [14] studies. This could be because of this study's particular focus on non-commercial chicken care (versus just small-scale) and especially on ex-commercial chicken carers. This study did not ask about other reasons for killing (e.g., due to illness or unwanted males); however, it was found that 98.8% of respondents would still look after hens in their care if they ceased laying eggs.

Of the 6.4% killing their chickens for consumption, cervical dislocation was the killing method selected by 57.3% of respondents, compared to decapitation by 11.5%, and killing cone (chicken is restrained upside down with the head extending through the narrow end of a cone-shaped device, allowing the neck to be clamped for dislocation) by 9.9%. A further 13.0% selected a mix of all stated methods and 8.4% selected "other," which predominantly comprised "broomstick method," captive bolt device, and mechanical dispatcher. A similar percentage of respondents used cervical dislocation in Correia-Gomes and Sparks' [14] study; however, in contrast, Elkhoraibi et al. [24] found the killing cone method to be the most common method utilized by respondents (34.1%). In this and other studies, respondents were not asked about stunning prior to attempts at killing; survey respondents should be asked explicitly about this in subsequent research to avoid assumptions as there are products available for stunning and awareness is being raised about them [82]. However, welfare concerns remain as backyard chicken keepers will likely be untrained in the proper use of both stunning and killing equipment. Stunning is also mentioned by a small proportion of respondents in the qualitative answers in this study. For private consumption purposes, it is not a legal requirement to stun chickens prior to using the cervical dislocation killing method in the UK and EU, though the Humane Slaughter Association recommends stunning beforehand [83], and it is illegal to decapitate chickens without prior stunning [84].

Relatedly, it is also concerning that amongst the qualitative responses, there was evidence of some chicken owners thinking that killing the chickens themselves is more humane than euthanasia by the vet. For some, this seems to align with the self-sufficiency and sovereignty ethos – one key motive for some chicken carers to keep chickens. The concern here is that owners are normally untrained, and there is no oversight. Attention should also be drawn to the fact that there is likely abuse of the term "euthanasia" amongst an extreme minority of non-commercial chicken owners. For instance, this is even evident amongst chicken scholars [17; p.280]. In this case, Zoubek [17] refers to the caring-killing paradox, which can be true for this minority of chicken owners. However, she then compares it to euthanasia in shelters of unwanted dogs. This comparison arguably fails as 1) dogs are not subsequently eaten, 2) such killing may actually be in some dogs' best interests as they may experience too poor levels of welfare if they reside in shelters very long term (true euthanasia requires that killing be in the individual's best interests), 3) it would be practiced by a vet, hence more likely to be humanely conducted (true euthanasia also requires that killing be humanely conducted). Increased availability of mobile avian-experienced vets would also remove concerns about stressful car journeys for chickens.
